# DUSP12 promotes cell cycle progression and protects cells from ZNF622 mediated apoptosis

**DOI:** 10.1038/s41419-026-08618-z

**Published:** 2026-03-18

**Authors:** Mai Abdusamad, Xiao Guo, Ivan Ramirez, Erick F. Velasquez, Whitaker Cohn, Ankur A. Gholkar, Immy A. Ashley, Yennifer Delgado, Mehdi Bouhaddou, Julian P. Whitelegge, Robert Damoiseaux, Jorge Z. Torres

**Affiliations:** 1 Department of Chemistry and Biochemistry, University of California, Los Angeles, CA USA; 2 Pasarow Mass Spectrometry Laboratory, The Jane and Terry Semel Institute for Neuroscience and Human Behavior, David Geffen School of Medicine, University of California, Los Angeles, CA USA; 3 Molecular Biology Institute, University of California, Los Angeles, CA USA; 4 Department of Microbiology, Immunology, and Molecular Genetics, University of California, Los Angeles, CA USA; 5 Institute for Quantitative and Computational Biosciences, University of California, Los Angeles, CA USA; 6 Jonsson Comprehensive Cancer Center, University of California, Los Angeles, CA USA; 7 California NanoSystems Institute, Los Angeles, CA USA; 8 Department of Molecular and Medical Pharmacology, Los Angeles, CA USA

**Keywords:** Cell biology, Cancer, Biochemistry, Cancer

## Abstract

Protein phosphatases are critical for regulating cell signaling, cell cycle, and cell fate decisions, and their dysregulation leads to an array of human diseases like cancer. The dual specificity phosphatases (DUSPs) have emerged as important factors driving tumorigenesis and cancer therapy resistance. DUSP12 is a poorly characterized atypical DUSP widely conserved throughout evolution. Although no direct substrate has been firmly established, DUSP12 has been implicated in protecting cells from stress, regulating ribosomal biogenesis, and modulating cellular DNA content. In this study, we used affinity- and proximity-based biochemical purification approaches coupled to mass spectrometry to identify the zinc finger protein ZNF622 as a novel DUSP12 interactor, which was validated by in cell and in vitro IP assays. Interestingly, ZNF622 binds to the unique zinc-binding domain of DUSP12, which previous reports indicated was important for many of DUSP12’s functions within the cell. Prior studies had implicated ZNF622 as a modulator of apoptosis, but it remained unclear if and how ZNF622 participated in the cell cycle and, more so, how it promoted cell death. Using mass spectrometry analyses, we found that overexpression of DUSP12 promoted de-phosphorylation of ZNF622 at Ser^143^. Overexpression of ZNF622, but not Ser^143^ phosphomimetic and phosphorylation-deficient mutants, led to an increase in pre-metaphase mitotic defects while knockdown of DUSP12 also showed mitotic defects in metaphase. Furthermore, knockdown of DUSP12 promoted, while knockdown of ZNF622 suppressed, stress-induced apoptosis. Our results support a model where DUSP12 protects cells from ZNF622 mediated stress-induced apoptosis.

## Introduction

During tumorigenesis, quality control measures that supervise the fidelity of the cell cycle are often disrupted, leading to abnormal cell growth [1]. In normal cells, cell cycle checkpoints are critical monitors of proper cell cycle progression and are, in turn, regulated by a complex network of signaling pathways [1]. Among these mechanisms, protein phosphorylation serves an important role as a molecular switch to regulate events in signaling pathways that determine a cell’s fate, including cell cycle progression, cell division, and cell death [2, 3]. While the regulatory role of kinases is well-established, less appreciated is the contribution of phosphatases in governing cell cycle progression [4].

Dual-specificity phosphatases (DUSPs), characterized by their unique ability to de-phosphorylate both tyrosine and threonine/serine residues within one substrate, are critical players of cell growth, survival, and death [5, 6]. DUSPs de-phosphorylate and down-regulate the activity of mitogen-activated protein kinases (MAPKs) and phosphatidyl inositol 3-kinases (PI3Ks), which implies a role in regulating tumorigenesis through these signaling pathways [5, 6]. Consistently, the dysregulation of DUSP activity is oncogenic or tumor-suppressive depending on the type of tumor [5]. For example, DUSP1 was found to be overexpressed in pancreatic cancer and its downregulation decreased tumor formation in a pancreatic cancer mouse model [7]. Similarly, the upregulation or overexpression of DUSP6 drives glioblastoma tumor formation in a glioblastoma mouse model [8]. Therefore, as illustrated by the growing list of proliferative malignancies associated with deregulation of DUSP expression, DUSP phosphatases represent exciting new targets for study [5, 6].

DUSP12 is a member of the atypical DUSPs, named for their vastly diverse substrate specificity and function, and is highly conserved across mammalian species [5, 9]. Several studies have linked aberrant expression of DUSP12 to the development of a wide range of cancers, highlighting the importance of investigating the biological activities of DUSP12 as dissecting its functions could provide insight into novel mechanisms supporting uncontrolled cell growth [10–14]. Within atypical DUSPs, DUSP12 is unique in that it contains a C-terminal cysteine-rich zinc-binding domain (ZBD) in addition to an N-terminal phosphatase domain [15]. Previous reports suggested that the ZBD mediates many of the observed effects of DUSP12 in the cell, including contributions to ribosome biogenesis, cell cycle progression, and cell survival [16–18]. However, the physiological role of DUSP12 remains unclear.

In this study, we determined that DUSP12 physically and functionally interacts with zinc finger protein 622 (ZNF622), also known as zinc finger protein 9 (ZPR9). ZNF622 has broad functions in the cell and has been linked to the regulation of cell growth, apoptosis, gene expression, and cell stress responses [19–25]. For example, ZNF622 binds to the B-MYB transcription factor to upregulate its transcriptional activity and modulate cell growth [19]. In hematopoietic stem cells, ZNF622 is important for 60S ribosome subunit biogenesis and inhibits the premature joining of the 60S and 40S subunits until needed, thereby regulating ribosome assembly, translation, and protein expression [20]. Interestingly, ZNF622 was found to co-purify with the ASCC2 subunit of the alkylation repair complex ASCC (activating signal cointegrator complex) after cells were exposed to the DNA-damaging agent MMS, suggesting that it may have a link to DNA repair [26]. Previous studies also showed that ZNF622 mediates apoptotic cell death through direct interaction with and activation by MPK38 and ASK1 in a phosphorylation-dependent manner [21–25]. Due to the role of ZNF622 in cell death, we sought to determine the relationship between DUSP12 and cell death. We demonstrate that the C-terminal zinc-binding domain of DUSP12 is important for binding to ZNF622, overexpression of DUSP12 promotes ZNF622 dephosphorylation and suppresses ZNF622 mediated apoptosis, and protects cells from cytotoxic agent stress-induced cell death.

## Results

### DUSP12 is important for cell division and cell cycle progression

**Fig. 1:**
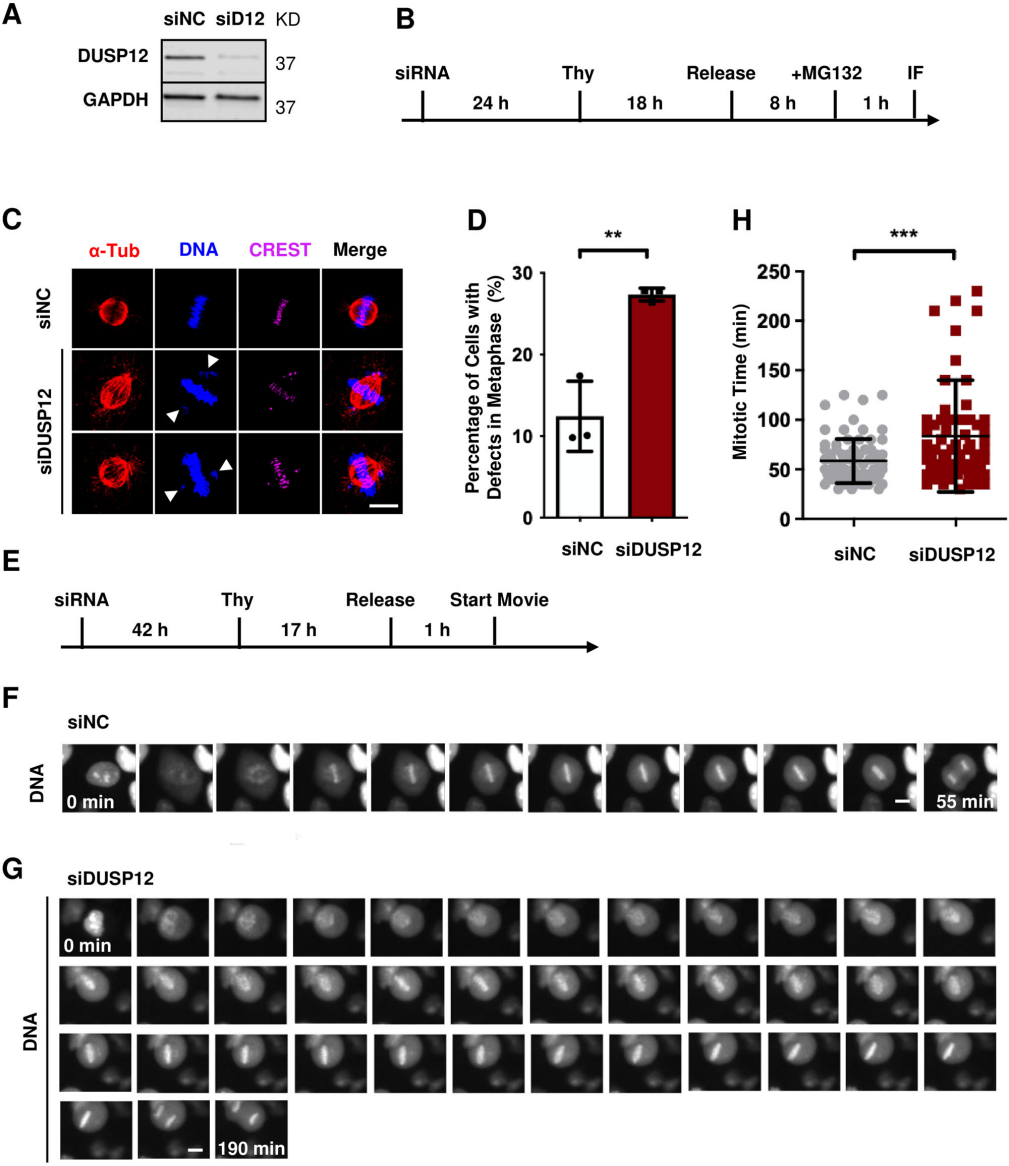
Knockdown of DUSP12 leads to mitotic defects. **A** Immunoblot analysis of protein extracts from HeLa cells transfected with non-targeting control siRNA (siNC) or siRNA targeting DUSP12 (siD12) for 72 h using anti-DUSP12 and anti-GAPDH antibodies. **B** Schematic of experiment performed in (**C**). **C** Knockdown of DUSP12 leads to chromosome misalignment in metaphase. HeLa cells were treated with siNC or siDUSP12 before fixation and co-staining with anti-CREST antibodies to visualize kinetochores and anti-α-tubulin antibodies to visualize the mitotic spindle, and the DNA dye Hoechst 33342. **D** Quantification of cells with misaligned, non-congressed chromosomes in metaphase (y-axis) for conditions shown in (**C**) (x-axis). **E** Schematic of live-cell time-lapse microscopy experiment performed in (**F**) and (**G**). **F**, **G** Depletion of DUSP12 leads to a slowed mitosis. Live-cell time-lapse microscopy of HeLa cells treated with siNC (**F**) or siDUSP12 (**G**) undergoing cell division. **H** Quantification of the timing of mitosis from chromosome condensation to chromosome segregation (y-axis) for conditions shown in (**F**) and (**G**) (x-axis). Scale bars: 10 μm. Data are shown as means ± SD. **p < 0.01, ***p < 0.001 (unpaired two-tailed Student’s t-test).

To evaluate the functional role of DUSP12 in cell division, we depleted HeLa cells of endogenous DUSP12 by RNAi (Fig. 1A) and performed a multiparametric analysis to monitor cell, chromosome, and mitotic spindle morphology during cell division by immunofluorescence (IF) microscopy (Fig. 1B). Knockdown of DUSP12 led to an increased percentage of cells with defects in chromosome alignment during metaphase (siNon-targeting Control (siNC) = 12.4 ± 6.5 and siDUSP12 = 27.4 ± 2.5, *p* < 0.01) (Fig. 1C, D). To examine the role of DUSP12 on mitotic duration, we coupled DUSP12 depletion in HeLa cells to live-cell time-lapse microscopy (Fig. 1E). This analysis showed that depletion of DUSP12 led to a significant increase in the time from chromosome condensation to chromosome segregation (siDUSP12 = 83.07 ± 54.46 min, *p* = 0.0004 compared with siNC = 58.47 ± 22.31 min) (Fig. 1F–H and Movie S1, 2).

In addition to the mitotic defects observed upon DUSP12 depletion, a significant difference in the cell cycle profile was observed (Supplementary Fig. S1A–F). Flow cytometry analysis showed that knockdown of DUSP12 increased the percentage of cells in G2/M phase compared to the control (Supplementary Fig. S1A, B, and S2A–F). A similar increase in mitotic defects and percentage of cells in G2/M phase was observed when DUSP12 was depleted with a second siRNA (siDUSP12-2) (Supplementary Fig. S3A–E, S4A–F). To further understand how this affected cell cycle progression, we coupled HeLa fluorescence ubiquitination cell cycle indicator (FUCCI) cells, which exhibit color changes according to the phase of the cell cycle, to live-cell time-lapse IF microscopy. HeLa FUCCI cells were depleted of endogenous DUSP12 by RNAi (Supplementary Fig. S1C, D). Cells were then synchronized with thymidine at G1/S phase and released into fresh media. Cells were imaged live four-hours post-release for 24 h. Both control and DUSP12-depleted conditions started at late-S/G2/M phase 4 h post-thymidine release. However, control cells continued to progress to G1 phase at 13 h post-thymidine release, while DUSP12-depleted cells lagged in late-S/G2/M (Supplementary Fig. S1E, F). This lag in DUSP12-depleted cells persisted even 28 h post-thymidine release (Supplementary Fig. S1E, F). Together, these results suggested that DUSP12 depletion led to a slowing of cell division.

### DUSP12 protein-protein interaction and protein proximity networks identify ZNF622 as a novel interactor

**Fig. 2:**
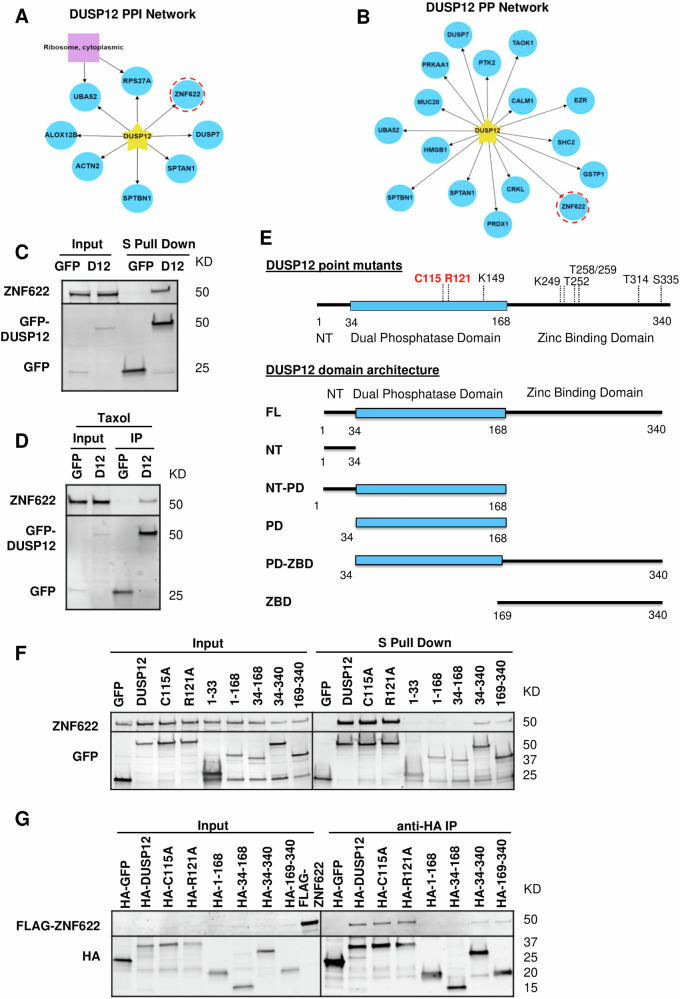
DUSP12 binds to ZNF622 via its C-terminal Zinc Binding Domain. **A**, **B** Summary of HeLa cell LAP-DUSP12 (**A**) and BioID2-DUSP12 (**B**) biochemical purifications and mass spectrometry analyses. ZNF622 was identified to interact with LAP-DUSP12 by protein-protein interaction (PPI) (**A**) and BioID2-DUSP12 protein proximity (**B**) networks using MAPK GO and CORUM complex annotation analyses. Yellow Star, bait protein DUSP12; Blue Circles, potential interactors; Purple Square, protein complex; Red Dashed Circles, ZNF622. **C**, **D** DUSP12 immunoprecipitates (IPs) with ZNF622. The LAP-DUSP12 HeLa stable cell line was induced to express LAP-DUSP12 in either asynchronized (**C**) or mitotic Taxol-arrested (**D**) cell populations. Cells were then harvested and LAP-DUSP12 IPs were analyzed by immunoblot. **E** Schematic of DUSP12 domain architecture. DUSP12 catalytic sites (C115, R121) are in *red*. FL full-length, NT N-terminus, NT-PD N-terminus and phosphatase domain, PD phosphatase domain, PD-ZBD phosphatase domain and zinc-binding domain, ZBD zinc-binding domain. **F**, **G** the ZNF622-DUSP12 interaction is dependent on the zinc binding domain of DUSP12. In (**F**) LAP-only, LAP-DUSP12-WT, LAP-C115A, LAP-R121A, and LAP-DUSP12-truncation HeLa stable cell lines were induced before being harvested for S-tag pull-downs and immunoblot analyses. In (**G**) FLAG-ZNF622, HA-DUSP12-WT, HA-C115A, HA-R121A, HA-DUSP12-truncations and HA-GFP (negative control) were expressed in vitro and incubated with anti-HA magnetic beads in IP assays.

To further define DUSP12’s cellular role, we sought to determine its protein interactome in Taxol-arrested mitotic cells. We established inducible localization and affinity purification (LAP = EGFP-TEV-S-Peptide)-tagged and biotin identification 2 (BioID2)-tagged DUSP12 HeLa stable cell lines, which were used to express LAP-/BioID2-DUSP12. These tagged versions of DUSP12 showed a similar subcellular localization to endogenous DUSP12, slightly enriched in the nucleus during interphase and on the mitotic spindle during mitosis (Supplementary Fig. S5A–D). LAP affinity and BioID2 proximity biochemical purifications were then carried out and the eluates were analyzed by LC-MS/MS. The mass spectrometry data was analyzed and visualized as protein interaction/association networks with CANVS software using the mitogen-activated protein kinase Gene Ontology (GO) terms (Supplementary Fig. S6A–C) [27]. Both the interaction and proximity networks identified ZNF622 (aka ZPR9) as a novel DUSP12 interaction/association (Fig. 2A, B). To validate the DUSP12-ZNF622 interaction, we performed LAP-DUSP12 immunoprecipitations (IPs) and confirmed that ZNF622 co-IP’d with DUSP12 in both asynchronous cells (Fig. 2C) and Taxol-arrested mitotic cells (Fig. 2D) by immunoblot analysis.

### The DUSP12 zinc-binding domain is important for binding to ZNF622

As the zinc-binding domain (ZBD) is important for many of DUSP12’s cellular functions, including its role in cell cycle regulation, we sought to determine whether the DUSP12 ZBD was important for binding to ZNF622. To do this in an unbiased manner, we generated a series of DUSP12 truncations (full length, N-terminus, N-terminus + phosphatase domain, phosphatase domain, phosphatase domain + C-terminus, and C-terminus) and catalytically dead mutants (C115A or R121A) (Fig. 2E and Supplementary Fig. S7A–D). We then developed a series of LAP-DUSP12 inducible stable cell lines capable of expressing these DUSP12 truncations and mutations. Next, we performed a series of IPs to investigate which domains of DUSP12 were involved in ZNF622 binding. These IPs showed that the N-terminus and the phosphatase domain of DUSP12 both failed to associate with ZNF622 (Fig. 2F). Whereas, DUSP12 full length, C-terminus, C-terminus + phosphatase domain, and the C115A and R121A mutants all pulled down ZNF622 (Fig. 2F). These results suggested that the C-terminus of DUSP12 was important for interacting with ZNF622. To further verify this, we performed in vitro binding assays with FLAG-tagged ZNF622 and HA-tagged DUSP12 full-length, truncations, and catalytic dead mutants. Similar results were observed, where ZNF622 co-IP’d with DUSP12 full length, C-terminus, C-terminus + phosphatase domain, and the C115A and R121A mutants (Fig. 2G). Taken together, our results indicated that the DUSP12 C-terminus (amino acids 169-340), which contained the ZBD, was critical for binding to ZNF622.

### Overexpression of ZNF622 leads to mitotic defects

Since DUSP12 interacted with ZNF622 during mitosis (Fig. 2D), we evaluated whether ZNF622 was important for cell division and, further, how this related to its interaction with DUSP12. Immunoblot analyses showed that endogenous ZNF622 was expressed constitutively throughout the cell cycle, which was similar to the DUSP12 expression profile (Supplementary Fig. S8A–D). Consistent with the finding that DUSP12 and ZNF622 interacted during cell division, IF microscopy showed that they shared similar localization patterns during mitosis (Supplementary Fig. S8E). More specifically, endogenous ZNF622 co-localized weakly to mitotic spindles during prometaphase and metaphase (Supplementary Fig. S8F, G).

**Fig. 3:**
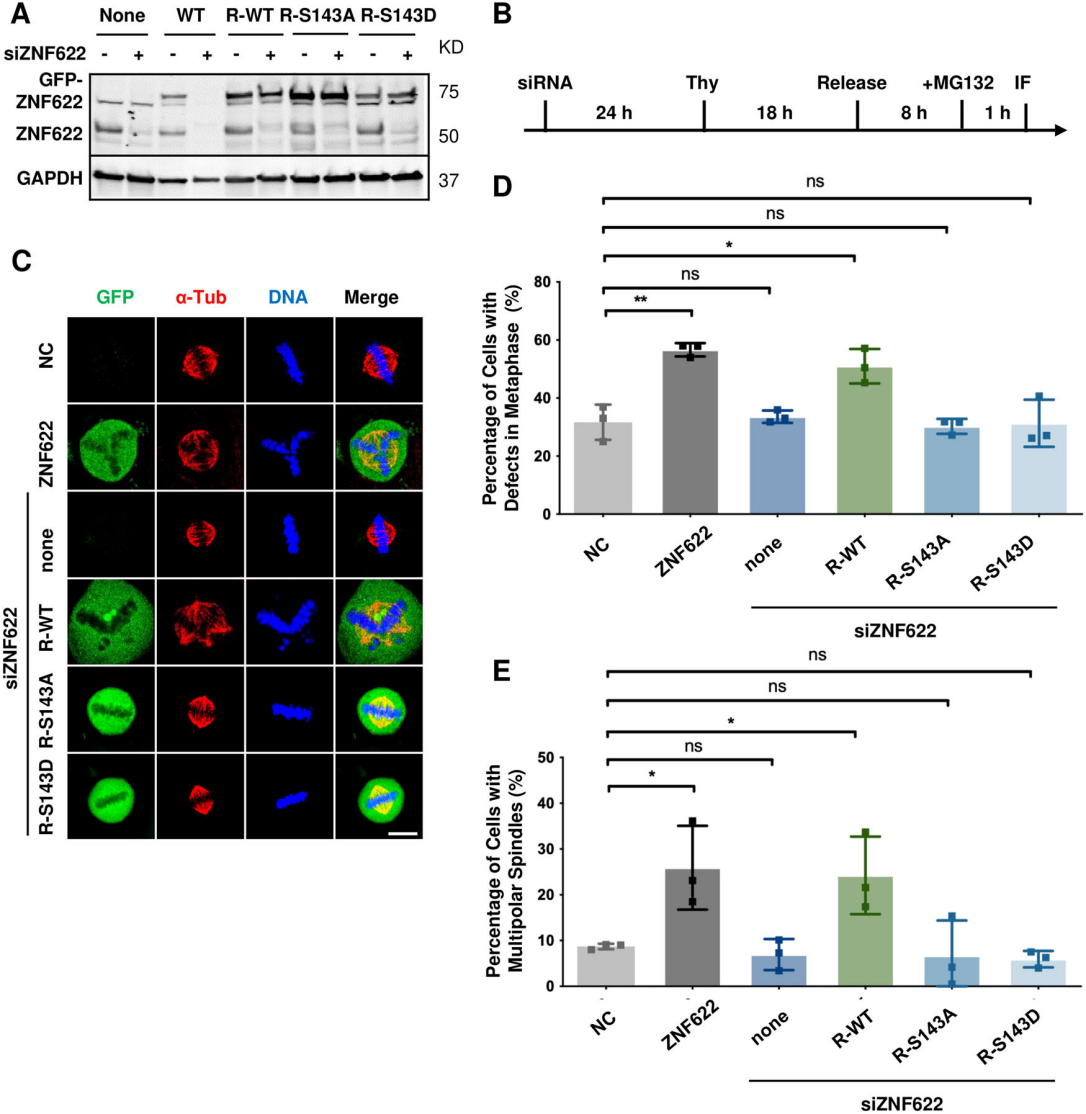
Overexpression of ZNF622 leads to mitotic defects. **A** Immunoblot analysis of HeLa cells induced to overexpress siRNA-sensitive wild-type ZNF622 (WT) or siRNA-resistant ZNF622 mutants (R-WT, R-S143A, or R-S143D) following transfection with or without ZNF622-targeting siRNA (siZNF622) for 72 h. **B** Schematic of experiments performed in (**C**). **C** Overexpression of ZNF622 wild type, but not siRNA resistant phospho-site mutants, leads to mitotic defects in metaphase. The LAP-ZNF622-WT and LAP-R-ZNF622 mutants (resistant to siRNA targeting ZNF622) HeLa inducible stable cell lines were transfected with siZNF622 for 24 h, synchronized in G1/S with thymidine for 18 h, released into the cell cycle for 8 h, treated with MG132 for 1 h, and induced with Dox during the last 16 h of the experiment to overexpress either GFP-ZNF622-WT or GFP-R-ZNF622 mutants. Cells were then fixed and stained with anti-GFP, Hoechst 33342 DNA dye, and anti-α-Tubulin antibodies. **D**, **E** Quantification of total cells with metaphase defects (**D**) and specifically those with multipolar spindles (**E**) (y-axis) for conditions shown (**C**) (x-axis). Scale bar: 10 μm. Data are shown as means ± SD. *p < 0.05, **p < 0.01 (unpaired two-tailed Student’s t-test).

To further assess the role of ZNF622 in cell division, we generated a LAP-ZNF622 inducible stable cell line to analyze the consequences of ZNF622 overexpression on mitotic progression by IF microscopy (Fig. 3A–C). Overexpression of ZNF622 led to a pronounced increase in the percentage of mitotic cells with defects in metaphase, such as non-congressed chromosomes and multipolar spindles (ZNF622 OE = 56.6 ± 2.3, *p* < 0.05 compared to the negative control (NC) = 31.7 ± 6.1) (Fig. 3D). This included a significant increase in mitotic cells with malformed spindles (multipolar and unfocused) (ZNF622 OE = 25.9 ± 9.1, *p* < 0.05 compared to NC = 8.7 ± 0.6) (Fig. 3C, E).

Given DUSP12’s capacity as a phosphatase, we next asked if DUSP12 modification of ZNF622 influenced cell division. LAP-GFP or LAP-DUSP12 stable cell lines were induced to overexpress these proteins, and ZNF622 was immunoprecipitated and analyzed by mass spectrometry (Supplementary Fig. S9A–C). This analysis revealed S143 as a potential site of de-phosphorylation on ZNF622 by DUSP12. Next, we performed a global phosphorylation analysis with cells overexpressing LAP-GFP or LAP-DUSP12, which showed that the level of ZNF622 S143 phorphorylation decreased when LAP-DUSP12 was overexpressed (Supplementary Fig. S9D). To evaluate the functionality of the identified phosphorylation site on ZNF622, phosphorylation-deficient (S/A) and phosphomimetic (S/D) mutations were introduced into LAP-ZNF622 (Supplementary Fig. S7E–G), and their effects were analyzed by IF. In cells depleted of endogenous ZNF622 by RNAi, overexpression of RNAi resistant wild type ZNF622 led to mitotic defects, whereas overpression of RNAi resistant ZNF622-S143 phosphosite mutants did not (Fig. 3C–E). Together, these results indicated that the dynamic oscilation between the phosphorylated and dephosphorylated state of ZNF622 S143 was important for a productive mitotis.

### DUSP12 protects cells from ZNF622 mediated cell death

**Fig. 4:**
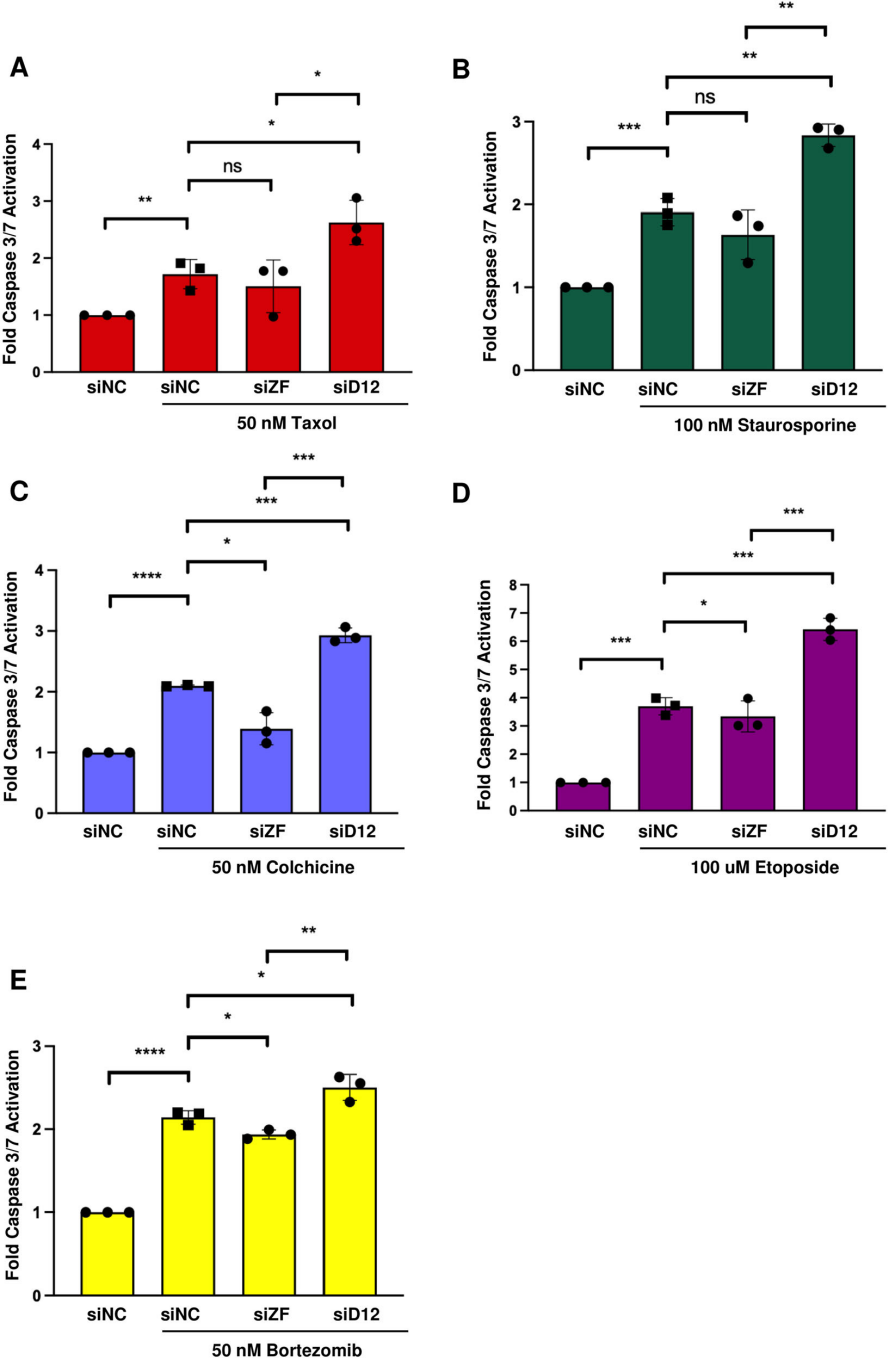
Knockdown of DUSP12 promotes, while knockdown of ZNF622 suppresses, stress induced apoptosis. HeLa cells were transfected with non-targeting control siRNA (siNC) or siRNA targeting ZNF622 (siZF) or DUSP12 (siD12) for 48 h, followed by treatment with 50 nM Taxol for 24 h (**A**), 100 nM Staurosporine for 24 h (**B**), 50 nM Colchicine for 24 h (**C**), 100 µM Etoposide for 24 h (**D**), or 50 nM Bortezomib for 24 h (**E**). Caspase 3/7 cleavage was then measured and expressed as fold change relative to control (y-axis) for the indicated conditions (x-axis). Data are shown as means ± SD. *p < 0.05, **p < 0.01, ***p < 0.001 ****p < 0.0001 (unpaired two-tailed Student’s t-test).

**Fig. 5:**
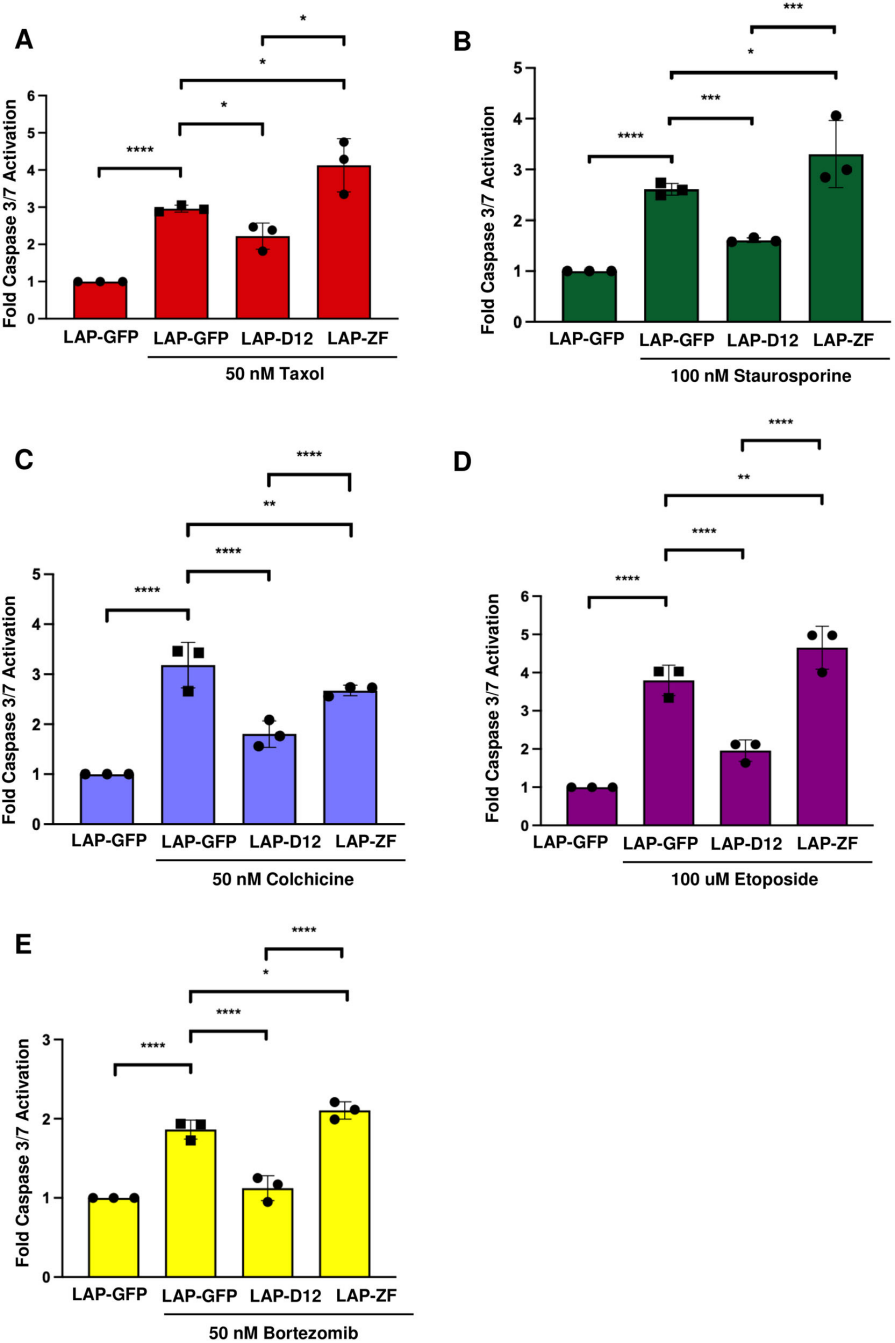
Overexpression of DUSP12 suppresses, while overexpression of ZNF622 promotes, stress induced apoptosis. LAP-GFP, LAP-DUSP12, and LAP-ZNF622 HeLa stable cell lines were induced to express these proteins with Dox and treated with 50 nM Taxol for 24 h (**A**), 100 nM Staurosporine for 24 h (**B**), 50 nM Colchicine for 24 h (**C**), 100 µM Etoposide for 24 h (**D**), or 50 nM Bortezomib for 24 h (**E**). Caspase 3/7 cleavage was then measured and expressed as fold change relative to control (y-axis) for the indicated conditions (x-axis). Data are shown as means ± SD. *p < 0.05, **p < 0.01, ***p < 0.001 ****p < 0.0001 (unpaired two-tailed Student’s t-test).

As both DUSP12 and ZNF622 had been separately linked to cell death, we sought to investigate their relationship as it relates to cell death in response to stress agents. HeLa cells depleted of DUSP12 or ZNF622 by RNAi were subjected to a panel of five chemotherapeutic and cytotoxic agents (Fig. 4A–E and Supplementary Fig. S10A–E). We then monitored apoptosis by the Caspase-Glo 3/7 assay. Depletion of DUSP12 led to a significant increase in apoptosis for all five treatments tested (Taxol, Staurosporine, Colchicine, Etoposide, and Botezomib), while depletion of ZNF622 led to a decrease in apoptosis in cells exposed to Colchicine, Etoposide, and Bortezomib (Fig. 4A–E and Supplementary Fig. S10A–E). Additionally, we analyzed the effect of depleting DUSP12 or ZNF622 on the apoptotic reponse of HEPG2 cells to Taxol, Staurosporine, and Etoposide. Depletion of DUSP12 led to a significant increase in apoptosis for all three treatments, while depletion of ZNF622 led to a decrease in apoptosis for all three treatments (Supplementary Fig. S11A–F). Consistently, a flow cytometric analysis of HeLa cells treated with Taxol showed that depletion of DUSP12 led to a significant increase in the percentage of Annexin V+ cells, while depletion of ZNF622 led to a decrease in Annexin V+ cells (Supplementary Fig. S12A). Next, we analyzed the effect of overexpressing LAP-DUSP12 or LAP-ZNF622 on apoptosis in HeLa cells. Overexpression of LAP-DUSP12 (shown to rescue the siDUSP12 apoptosis phenotype, Supplementary Fig. S13) led to a significant decrease in apoptosis in response to all five treatments (Taxol, Staurosporine, Colchicine, Etoposide, and Botezomib), while overexpression of LAP-ZNF622 led to a significant increase in apoptosis in response to four treatments (Taxol, Staurosporine, Etoposide, and Botezomib), but not Colchicine (Fig. 5A–E and Supplementary Fig. S14A–E). These results indicated that ZNF622 promoted and DUSP12 inhibited stress-induced apoptosis in response to most chemotherapeutics and ctotoxic agents.

**Fig. 6:**
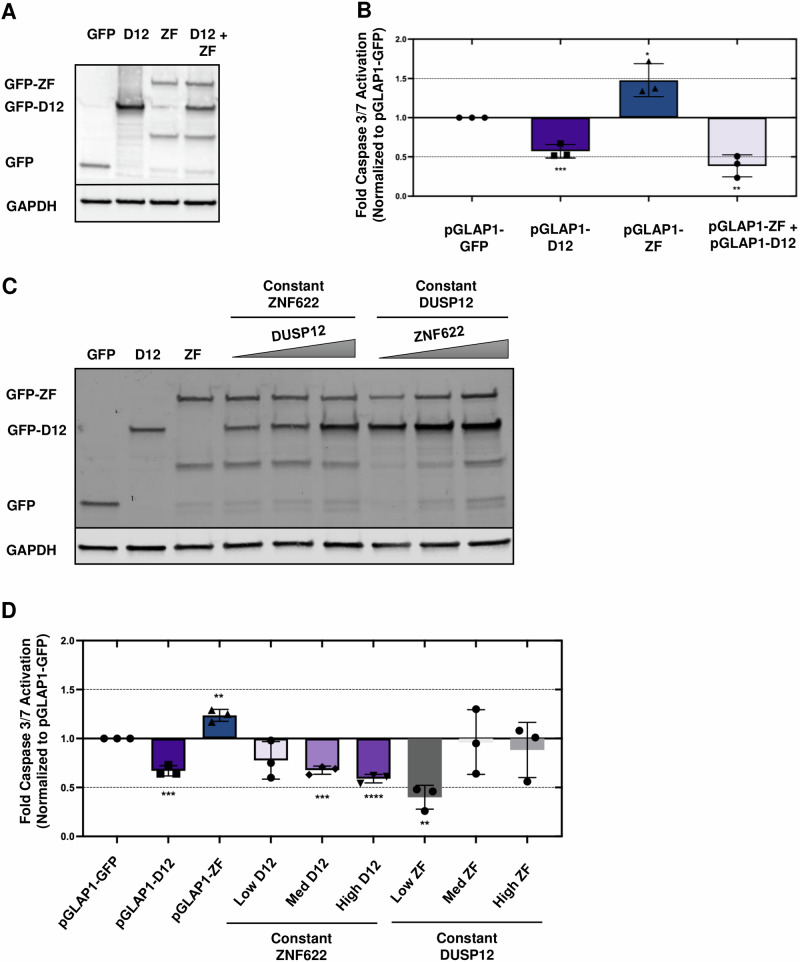
DUSP12 and ZNF622 exert opposing, level-dependent effects on apoptosis. **A** HeLa cells were transiently transfected with pGLAP1-GFP, pGLAP1-DUSP12, pGLAP1-ZNF622, or pGLAP1-DUSP12 + pGLAP1-ZNF622 for 24 h and their expression was monitored by immunoblot analysis. **B** Caspase 3/7 cleavage was then measured and expressed as fold change relative to control (y-axis) for the indicated conditions (x-axis). **C**, **D** Increasing DUSP12 expression decreased apoptosis, whereas increasing ZNF622 expression enhanced apoptosis, demonstrating opposing, dose-dependent effects. **C** HeLa cells were co-transfected with varying amounts of DUSP12 or ZNF622 while keeping the other protein constant for 24 h. **D** Caspase 3/7 cleavage was then measured and expressed as fold change relative to control (y-axis) for the indicated conditions (x-axis). Data are shown as means ± SD. *p < 0.05, **p < 0.01, ***p < 0.001, ****p < 0.0001 (unpaired two-tailed Student’s t-test).

Next, we analyzed whether DUSP12 could suppress ZNF622 mediated cell death without the presence of cytotoxic agents using the the Caspase-Glo 3/7 apoptosis assay. First, HeLa cells were transiently transfected with LAP-DUSP12 or LAP-ZNF622 for 24 h to evaluate proxy basal levels of cell death (Fig. 6A, B). While introduction of LAP-DUSP12 led to lower levels of cell death compared to the control, expression of LAP-ZNF622 led to an increase in apoptotic cell death (Fig. 6A, B). Meanwhile, co-expression of LAP-ZNF622 and LAP-DUSP12 led to a marked reduction in apoptotic cell death that was comparable to LAP-DUSP12 alone (Fig. 6A, B). Similarly, flow cytometry analysis of HeLa cells showed that overexpression of LAP-DUSP12 led to a significant decrease in the percentage of Annexin V+ cells, overexpression of LAP-ZNF622 led to an increase in Annexin V+ cells, and co-expression of LAP-ZNF622 and LAP-DUSP12 led to a reduction in Annexin V+ cells that was comparable to LAP-DUSP12 alone (Supplementary Fig. S12B). Next, we held the expression levels of LAP-ZNF622 constant and increased the levels of LAP-DUSP12 (low, medium, and high) and measured the levels of apoptosis using the Caspase-Glo 3/7 assay (Fig. 6C, D). This showed that increasing the concentrations of LAP-DUSP12 led to decreasing levels of apoptosis (Fig. 6C, D). Next, we held the expression levels of LAP-DUSP12 constant and increased the levels of LAP-ZNF622 (low, medium, and high) (Fig. 6C, D). This showed that medium and high levels of LAP-ZNF622 increased the levels of apoptosis compared to low levels of LAP-ZNF622 (Fig. 6C, D).

**Fig. 7:**
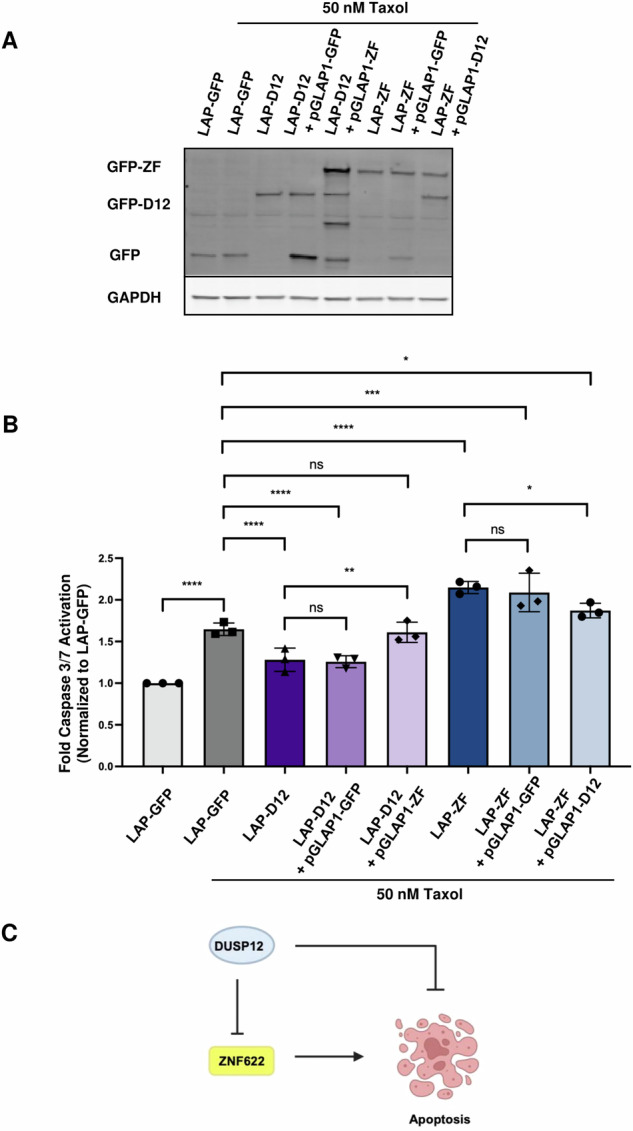
DUSP12 protects cells from ZNF622-driven apoptosis. **A** LAP-GFP, LAP-DUSP12, and LAP-ZNF622 HeLa stable cell lines were induced with Dox to express these proteins and transiently transfected with the indicated constructs for 4 h then treated with 50 nM Taxol for 24 h after. **B** Caspase 3/7 cleavage was then measured and expressed as fold change relative to control (y-axis) for the indicated conditions (x-axis). Data are shown as means ± SD. **C** Putative model of how DUSP12 protects cells from ZNF622-driven apoptosis. *p < 0.05, **p < 0.01, ***p < 0.001, ****p < 0.0001 (unpaired two-tailed Student’s t-test).

Next, we sought to investigate whether DUSP12 could also protect cells exposed to stress conditions in which ZNF622 had been shown to play a cytotoxic role. LAP-GFP, LAP-DUSP12, and LAP-ZNF622 HeLa stable cell lines were subjected to transfection with an empty vector, DUSP12, or ZNF622 and 50 nM Taxol for 24 h and analyzed using the the Caspase-Glo 3/7 assay (Fig. 7A, B). While expression of ZNF622 promoted a significant increase in Taxol mediated cell death, co-expression with DUSP12 ameliorated the cell death (Fig. 7B). Similarly, flow cytometry analysis of HeLa cells treated with Taxol showed that overexpression of DUSP12 led to a significant decrease in the percentage of Annexin V+ cells, overexpression of ZNF622 led to an increase in Annexin V+ cells and co-expression of LAP-ZNF622 and LAP-DUSP12 led to a reduction in Annexin V+ cells that was comparable to LAP-DUSP12 alone (Supplementary Fig. S12C). Together, these results indicated that DUSP12 mitigated ZNF622 mediated cell death (Fig. 7C).

## Discussion

This study advances our understanding of the factors and pathways that regulate cell cycle and cell death decisions. Our results are consistent with a model where DUSP12 binds to and counteracts the ability of ZNF622 to induce apoptosis in response to cytotoxic agents (Fig. 6C). We show that ZNF622 is a novel DUSP12 interacting partner and that this interaction relies on the DUSP12 zinc-binding domain. Further, DUSP12 promoted de-phosphorylation of ZNF622 at S143 and this modification was necessary to suppress ZNF622 mediated mitotic defects. Consistently, DUSP12 depletion led to mitotic defects and stress-induced cell death. Furthermore, overexpression of ZNF622 also led to an increase in cell death in response to most stress-inducers, but not Colchicine. A possible explanation for this difference in response could be that Colchicine synergizes with ZNF622 overexpression to trigger a fast cell death that is beyond the point of detection with the Caspase 3/7 activation detection assay. Another possibility is that Colchicine treatment depolymerizes mitotic spindles and mitigates the cell death that ZNF622 overexpression induces due to its ability to generate multipolar spindles (Fig. 3C). Interestingly, co-expression of ZNF622/DUSP12 mitigated ZNF622-induced apoptosis. Together, these results establish the molecular crosstalk between DUSP12 and ZNF622 in the regulation of cell cycle progression and cell death. It’s important to note that our mass spectrometry analyses with LAP-DUSP12 or BioID2-DUSP12 defined only one of eight binary interactions described in UniProt for DUSP12 (Eukaryotic translation initiation factor 5, EIF5). On the other hand, our analyses identified numerous novel DUSP12 interactions/associations that link to the regulation of cell cycle progression and apoptosis (Supplementary Fig. S6 and Supplementary Table S3, S4). Differences in experimental approaches in our study and previous studies can account for these differences. For example, our LAP-DUSP12 or BioID2-DUSP12 purifications were specifically performed with Taxol-arrested mitotic cells. In addition to arresting cells in mitosis, Taxol is a cytotoxic stress inducer that promotes apoptosis, a process DUSP12 helps suppress (Figs. 4, 5, 7, and Supplementary Figs. S11, S12, S13). Additionally, to our knowledge, this is the first study to use LAP- and BioID2-DUSP12 mass spectrometry analyses to define DUSP12 interactions/associations.

Our results also help explain previous cancer studies where DUSP12 was found to be amplified or up-regulated in several types of cancers including hepatocellular carcinoma, neuroblastomas, sarcomas, and retinoblastomas, which in many cases correlated with disease progression [10, 13, 14, 28, 29]. We show that DUSP12 overexpression protects cells from the cytotoxic effects of a set of chemotherapeutic agents, while DUSP12 depletion sensitizes cells to these agents. Therefore, DUSP12 is a promising target in cancers that overexpress DUSP12 or harbor DUSP12 amplification. The development of specific DUSP12 pharmacological inhibitors will be critical to determining if inhibition of DUSP12 activity can render these cancers susceptible to cytotoxic agents through the activation of apoptosis, and whether combination treatments prove to be more efficacious than single treatments. Therefore, it will be of interest to determine if DUSP12 overexpression protects against other common types of chemotherapeutic agents that induce apoptosis or if DUSP12 has roles in protecting cells from other forms of cell death like autophagy or necrosis [30].

On the other hand, protection against apoptosis is important in certain stress scenarios, such as the protection of tissues and organs during trauma-driven apoptosis [31]. Interestingly, recent studies have indicated that DUSP12 can protect against apoptosis in a hepatic ischemia-reperfusion injury mouse model [32, 33]. Therefore, the development of pharmacological activators of DUSP12 and/or inhibitors of ZNF622 could prove to be beneficial in ameliorating liver failure during liver surgery. This reasoning could also be expanded to diseases that are driven by apoptosis, like neurodegenerative disorders, where the inhibition of apoptosis could ameliorate disease progression [31]. Therefore, it will be of interest to determine the expression levels of DUSP12 and ZNF622 in these disease models and the importance of the DUSP12-ZNF622 interaction in regulating apoptosis.

## Materials and Methods

### Cell culture

Table S2 lists all reagents and tools used in this study. HEK-293T, HeLa, HeLa Flp-In T-Rex, and HEPG2 cells were cultured in DMEM/Ham’s F-12 with L-glutamine (Genesee Scientific, Morrisville NC) supplemented with 10% fetal bovine serum and 1% penicillin-streptomycin (Life Technologies, Carlsbad CA). Cells were incubated in 5% CO_2_ at 37 °C and passaged twice weekly using 0.25% Trypsin-EDTA (Life Technologies). Modified HeLa Flp-In T-Rex stable cell lines were generated as described below. Cells were tested for mycoplasma contamination using the MycoStrip mycoplasma detection kit (InvivoGen, San Diego CA).

### Cell synchronization, transfection, and inhibitor treatment

For G1/S arrest and release experiments, cells were treated with 2 mM thymidine (Sigma-Aldrich, St. Louis MO) for 18 h, washed three times with PBS, two times with complete media, and then released into fresh media. For G2/M arrests, cells were treated with 100 nM Taxol (Sigma-Aldrich) for 18 h. For metaphase arrests, cells were treated with 10 µM MG132 (Millipore Sigma, Burlington MA) for 1 h post-thymidine release. For siRNA experiments, cells were transfected with Silencer Select Validated siRNA (Thermo Fisher, Canoga Park CA) against DUSP12, ZNF622, or a non-targeting control using RNAiMAX (Thermo Fisher) as described previously [34]. For protein overexpression experiments, cells were transfected with 1 µg LAP-tagged DUSP12 or ZNF622 using FuGENE HD (Promega, Madison WI) for the indicated times according to the manufacturer’s instructions. For combination experiments with cytotoxic agents, cells were transfected and subsequently incubated with drugs for the indicated times.

### Plasmids, mutagenesis, and generation of stable cell lines

Site-directed mutagenesis using the QuikChange Lightning Site-Directed Mutagenesis Kit (Agilent, Santa Clara CA) was performed to generate DUSP12 and ZNF622 mutants. cDNAs of GFP, DUSP12, DUSP12 truncations, DUSP12 catalytically dead mutants, ZNF622, and ZNF622 phospho-site mutants were cloned into pGLAP1, pGBioID2, pCS2-HA, or pCS2-FLAG via Gateway LR Clonase (Thermo Fisher) reactions to generate N-terminally tagged fusion proteins. pGLAP1-only/DUSP12/DUSP12-C115A/DUSP12-R121A/DUSP12-truncations/ZNF622/ZNF622-S143A/ZNF622-S143D and pGBioID2-only/DUSP12 were used to generate doxycycline-inducible HEK-293T or HeLa Flp-In T-Rex LAP-GFP/DUSP12/DUSP12-C115A/DUSP12-R121A/DUSP12-truncations/ZNF622/ZNF622-S143A/ZNF622-S143D and BioID2-only/DUSP12 stable cell lines as described previously [35, 36]. HeLa cells were transduced with pLX304-3xNLS-EBFP2 lentiviral expression vector and sorted for high expression using a FACSAria III (BD Biosciences, Franklin Lakes NJ).

### LAP/BioID2 purifications and LC-MS/MS analyses

For LAP affinity purifications, Taxol-arrested LAP-tagged inducible stable cell lines were purified as previously described [35, 36]. Briefly, LAP-only and LAP-DUSP12 stable cell lines were induced with 0.1 µg/mL doxycycline (Sigma-Aldrich) and arrested in mitosis with 100 nM Taxol for 18 h before being harvested and lysed. Cleared lysates were subjected to tandem affinity purification by incubation with anti-GFP antibody beads, and the bound eluates were incubated with S-protein Agarose (Millipore Sigma). Final eluates were trypsinized for subsequent LC-MS/MS analysis. For BioID2 proximity purifications, biotinylated proteins were purified from Taxol-arrested BioID2-tagged inducible stable cell lines as previously described [34, 37]. Briefly, BioID2-only and BioID2-DUSP12 stable cell lines were pre-incubated with DMEM/Ham’s F-12 supplemented with 10% streptavidin Dynabead-treated (Thermo Fisher) FBS overnight at 4 °C. Cells were induced with 0.1 μg/mL Dox and treated with 100 nM Taxol and 50 μM Biotin for 16 h before being harvested and lysed. Cleared lysates were purified by incubation with Dynabeads overnight at 4 °C, and final eluates were trypsinized for downstream LC-MS/MS analysis. For mapping the dephosphorylation site on ZNF622 by DUSP12, LAP-only, LAP-ASK1, and LAP-DUSP12 were expressed with 0.1 µg/mL doxycycline for 18 h. The induced stable cell lines were harvested and lysed in LAP200 lysis buffer (50 mM Hepes pH 7.4, 200 mM KCl, 1 mM EGTA, 1 mM MgCl_2_, 10% glycerol) supplemented with 0.05% NP-40, 0.5 mM DTT and protease inhibitor cocktail (Thermo Fisher). Cell lysates were incubated with an anti-ZNF622 antibody (Santa Cruz Biotechnology, Dallas TX) coupled to Protein A-coupled Sepharose beads (Bio-Rad, Hercules CA) for 2 h before being prepared for LC-MS/MS analysis. Mass spectrometry analysis on all samples was performed on a Thermo Q Exactive Plus Orbitrap as described previously [34]. Protein-protein interaction (affinity) and protein-protein association (proximity) data were analyzed and visualized with CANVS [27]. CANVS integrated data from the Biological General Repository for Interaction Datasets (BioGRID v.3.5) [38] and the Comprehensive Resource of Mammalian Protein Complexes (CORUM v. 3.0) [39] to create networks that associated proteins based on cellular mechanisms by using Gene Ontology (GO) terms [40] and the RCytoscapeJS [41, 42] visualization package. The global phosphoproteomic analysis was carried out as described previously [43], with modificaitons to the volumes, see supplementary information for a detailed protocol. Mass spectrometry data was deposited in RPIDE (Proteomics IDEntifications Database, Project accession: PXD072946, Token:JAIFp2mNpb38).

### Microscopy

Cell fixation and microscopy were carried out as described previously [44]. Briefly, cells grown on glass coverslips in a 24-well plate were fixed with 4% paraformaldehyde and permeabilized with 0.2% Triton X-100/PBS. Subsequently, cells were blocked with IF buffer (1x PBS, 5% Fish Gelatin, 0.1% TritonX-100) before being stained with 0.5 µg/mL Hoechst 33342 and the indicated primary antibodies in IF buffer for 1 h at room temperature. After three PBS washes, cells were incubated with secondary antibodies in IF buffer for 30 min at room temperature and washed three times with PBS. Coverslips were mounted with ProLong Gold Antifade mounting solution (Thermo Fisher) on glass slides and sealed with nail polish. Representative cells were selected and imaged either on a Leica (Wetzlar Germany) DMI6000 microscope (63x/1.40 NA oil objective, Leica AF6000 Analysis Package), a Leica MICA microscope (63x/1.40 NA oil objective, MICA Analysis Package), or an ImageXpress XL imaging system (Molecular Devices) and exported as TIFF files. Primary and secondary antibodies and their corresponding information can be found in Supplementary Table S2. For live cell imaging, HeLa-3xNLS-EBFP2 cells were imaged live one hour post thymidine release for 17 h with an ImageXpress XL imaging system (Molecular Devices, San Jose CA) using a 20x air objective. Images were captured every five minutes with the DAPI channel and converted to AVI movies with ImageJ at one frame per second. HeLa FUCCI cells were imaged live four hours post-thymidine release with a Leica MICA microscope using a 20x air objective. Images were captured every ten minutes with FITC and Cy3 channels and exported as TIFF files.

### IPs and binding assays

For immunoprecipitation, LAP-tagged induced stable cell lines or transiently transfected HeLa cells were harvested and lysed, then incubated with S-protein Agarose (Millipore Sigma) for 2 h at 4 °C. After incubation, bound beads were washed three times with LAP100 buffer (50 mM Hepes pH 7.4, 100 mM KCl, 1 mM EGTA, 1 mM MgCl2, 10% glycerol) supplemented with 0.05% NP-40 and 0.5 mM DTT. For in vitro binding assays, HA-tagged GFP/DUSP12/DUSP12-C115A/DUSP12-R121A/DUSP12-truncations and FLAG-tagged ZNF622 were expressed in a Quick Coupled Transcription/Translation System (IVT) (Promega) and incubated together with anti-HA magnetic beads (MBL, Tokyo Japan) for 1.5 h at 4 °C. After incubation, bound beads were washed three times with LAP200 buffer (50 mM Hepes pH 7.4, 200 mM KCl, 1 mM EGTA, 1 mM MgCl2, 10% glycerol) supplemented with 0.05% NP-40 and 0.5 mM DTT. Final IP and IVT eluates were resolved by SDS-PAGE for immunoblot analysis.

### Immunoblotting

Cell protein extracts were prepared from cells grown in 6-well plates. After harvesting, cells were lysed in LAP200 lysis buffer (50 mM Hepes pH 7.4, 200 mM KCl, 1 mM EGTA, 1 mM MgCl_2_, 10% glycerol) supplemented with 0.3% NP-40, 1 µM DTT, Phosphatase Inhibitor and Protease Inhibitor Cocktail tablets (Thermo Fisher). Cell extracts were cleared by spinning at 15000 RPM for 10 min at 4 °C. Protein concentration was quantified by BCA analysis and 6x SDS reducing sample buffer was added. Processed total lysates were separated by SDS-PAGE and transferred to a PVDF membrane (EMD Millipore). Membranes were incubated in blocking buffer (PBS, 0.5% BSA, 0.05% Tween-20, 0.02% SDS, 0.05% Proclin) and then with the indicated primary and secondary antibodies to visualize protein levels. The imaging of immunoblots was performed with a LI-COR (Lincoln NE) Odyssey Imaging system. Full length uncropped original western blots can be found in Supplementary File 1.

### Caspase 3/7 cleavage activity

Cells were seeded (5000 cells/well for HeLa; 20,000 cells/well for HEPG2) in black, clear-bottom 96-well plates (Corning, Corning NY). Indicated transfections and/or drug treatments were added simultaneously or 24 h after plating. Apoptosis was measured at the indicated times following indicated treatment using the Caspase-Glo 3/7 Assay (Promega) as described previously [45]. Experiments were performed in triplicate, averaged and normalized to a negative-matched (non-targeting) control.

### DUSP12 flow cytometry

For cell cycle analyses, cells treated with the indicated siRNAs for 72 h and harvested using TrypLE Express (Thermo Fisher) and washed with PBS. Cold 70% ethanol was added to the cells dropwise, followed by incubation overnight at 4 °C. Fixed cells were centrifuged and stained with 400 µL PI/RNase (Thermo Fisher) solution per million cells. Cells were incubated for 15 min at 25 °C. Data acquisition was performed using the Attune NxT Flow Cytometer (Invitrogen). Data was analyzed using a ModFit (Cytonome Verity, Lexington MA) cell cycle analysis program, gating against smaller cellular debris as well as events consistent with more than one cell per droplet.

### Annexin and 7-AAD staining

To further evaluate the extent of cell death, cells were harvested following indicated combination treatment using TrypLE Express (Thermo Fisher), then stained for 15 min at room temperature with Annexin V- eFluor 450 (Thermo Fisher), washed with PBS, and then incubated for 15 min at 4 °C with 7-Aminoactinomycin D (7-AAD) (Thermo Fisher). Cells were then washed, and then all single cells in each sample were analyzed by flow cytometry. Data were analyzed using FlowJo (FlowJo LLC, Ashland OR), gating against smaller cellular debris as well as events consistent with more than one cell per droplet.

### Antibodies

Supplementary Table S2 lists all primary and secondary antibodies used in this study.

### Quantification and statistical analysis

For IF microscopy quantification, three independent experiments were performed with 100 cells counted per experiment (*n* = 300). For live cell time-lapse microscopy quantification, three independent experiments were performed for each condition with 25 cells counted per experiment (*n* = 75). All apoptosis assays were performed as three biological replicates with three technical replicates each. The data was analyzed using unpaired Student’s t-test in Figs. 1D, H, 3D, E, 4A–E, 5A–E, 6B, D, 7B, S1B, S1F, S2D, S2E, S11B, S11D, S11F, S12A**-**C, S13B, and S13D. *P*-values were calculated using an unpaired Student’s t-test between two groups. Data is judged to be statistically significant when *P* < 0.05. Asterisks indicate statistical significance as *P < 0.05, **P < 0.01, ***P < 0.001, and ****P < 0.0001. All data is presented as mean ± standard deviation. GraphPad Prism 10 (La Jolla CA) was used for statistical analysis. Aivia AI Image Analysis Software (Leica) was used to quantify cells at each cell cycle phase in Figure S1E. ModFit LT 6.0 was used to analyze the cell cycle profile in Figure S1F. Flow cytometry data from Annexin V/7-AAD staining were analyzed using FlowJo v11 to quantify apoptotic populations in Figure S12A–C.

## Supplementary information

Supplemental Material file

Full Uncropped Western Blots

Table S1

Table S2

Table S3

Table S4

Movie S1

Movie S1

## Data Availability

Additional source data is provided in the supplementary material. Other data that support the findings of this study are available from the corresponding author upon reasonable request. Mass spectrometry data was deposited in RPIDE (Acession: PXD072946, Token:JAIFp2mNpb38).
