# Supplementary material for: DUSP12 promotes cell cycle progression and protects cells from ZNF622 mediated apoptosis
Source: Cell Death Dis. 2026 Mar 18;17(1):315. doi: 10.1038/s41419-026-08618-z (PMC13039736; doi:10.1038/s41419-026-08618-z)
Supplement: Supplementary file 1 — Supplemental Material file [file 41419_2026_8618_MOESM1_ESM.pdf]

SUPPLEMENTARY INFORMATION

DUSP12 promotes cell cycle progression and protects cells from ZNF622 mediated apoptosis

Mai Abdusamad[1], Xiao Guo[1], Ivan Ramirez[1], Erick F. Velasquez[1], Whitaker Cohn[2], Ankur A. Gholkar[1], Immy A. Ashley[3,4,5], Yennifer Delgado[3,4,5], Mehdi Bouhaddou[3,4,5], Julian P. Whitelegge[2,3,6], Robert Damoiseaux[6,7,8], and Jorge Z. Torres[1, 3, 6*]

[1]Department of Chemistry and Biochemistry, University of California, Los Angeles, CA 90095, USA
[2]Pasarow Mass Spectrometry Laboratory, The Jane and Terry Semel Institute for Neuroscience and Human Behavior, David Geffen School of Medicine, University of California, Los Angeles, CA 90095, USA
[3]Molecular Biology Institute, University of California, Los Angeles, CA 90095, USA
[4]Department of Microbiology, Immunology, and Molecular Genetics, University of California, Los Angeles, CA 90095, USA
[5]Institute for Quantitative and Computational Biosciences, University of California, Los Angeles, CA 90095, USA
[6]Jonsson Comprehensive Cancer Center, University of California, Los Angeles, CA 90095, USA
[7]California NanoSystems Institute, Los Angeles, CA 90095, USA
[8]Department of Molecular and Medical Pharmacology, Los Angeles, CA 90095, USA

*Corresponding author:
Jorge Z. Torres
607 Charles E. Young Drive East
Los Angeles, CA 90095
Phone: 310-206-2092
jorget@ucla.edu

Table of contents: **Page**

**SUPPLEMENTARY FIGURES**	**S2 – S15**
**SUPPLEMENTARY MOVIE LEGENDS**	**S16**
**SUPPLEMENTARY TABLE LEGENDS**	**S16**
**SUPPLEMENTARY METHODS**	**S16**

SUPPLEMENTARY FIGURES

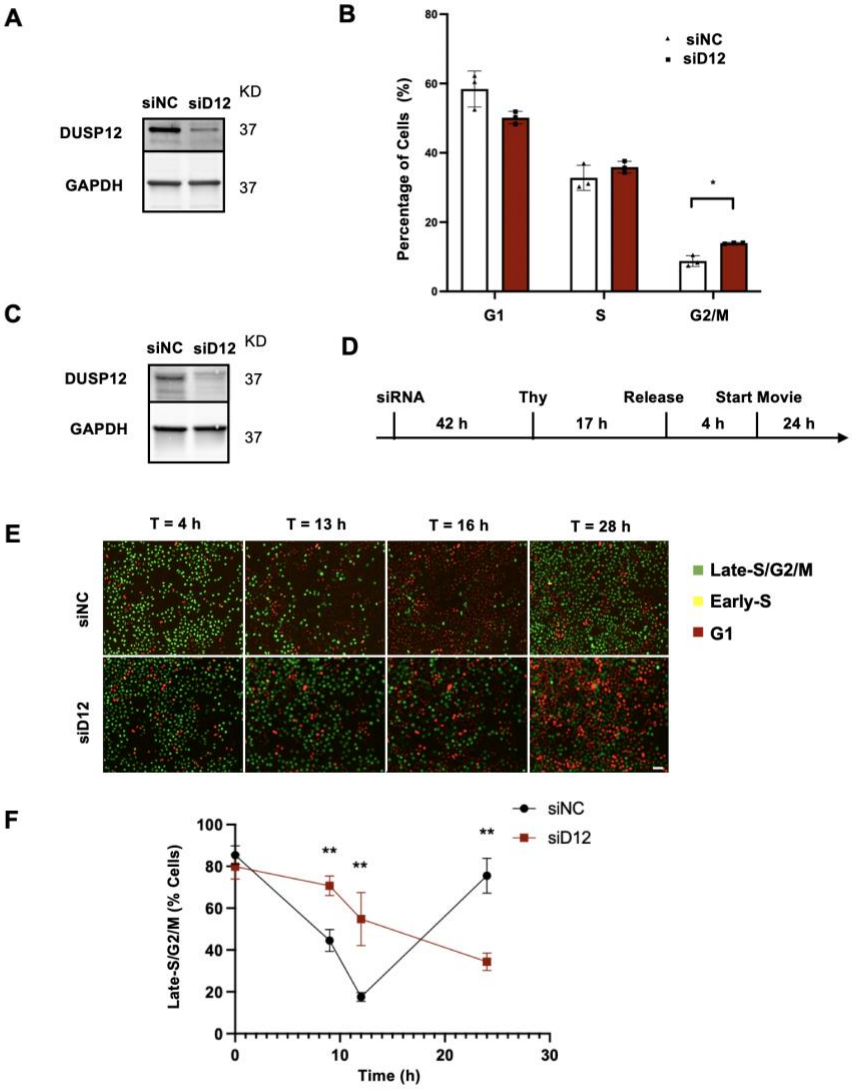

Figure S1. Knockdown of DUSP12 leads to mitotic slowing. A siRNA knockdown of endogenous DUSP12 in HeLa cells. Cells were transfected with non-targeting control (siNC) or DUSP12 siRNA (siD12) for 72 hours, followed by lysis and immunoblot analysis. **B** Flow cytometry cell cycle profiling of cells transfected with siNC or siDUSP12 for 72 h and stained with PI shows that DUSP12 depletion slows cells in G2/M phase. **C** siRNA knockdown of endogenous DUSP12 in HeLa FUCCI cells. Cells were transfected with siNC or siD12 for 72 hours, followed by lysis and immunoblot analysis. **D** Schematic of live-cell time-lapse microscopy experiment performed in (**E**). **E, F** Knockdown of DUSP12 leads to late-S/G2/M slowing. HeLa FUCCI cells were treated with siNC or siDUSP12 for 42 h, synchronized in G1/S with thymidine for 17 h, released into the cell cycle for 4 h, and then subjected to live-cell imaging for 24 h to monitor cell cycle progression. Representative images in (**E**) reflect time following release into the cell cycle (e.g. T = 4 h post-thymidine release). Cell cycle phases are indicated by color: G1 (red), early-S (yellow), or late-S/G2/M (green). Bar indicates 20 μm. (**F**) Quantification of cells in late-S/G2/M (y-axis) for conditions shown in (E) (x-axis). Cells at each cell cycle phase were quantitatively assessed using Aivia AI Image Analysis Software. Data are shown as means ± SD. *$p < 0.05$, **$p < 0.01$ (unpaired two-tailed Student's t-test). Related to Figure 1.

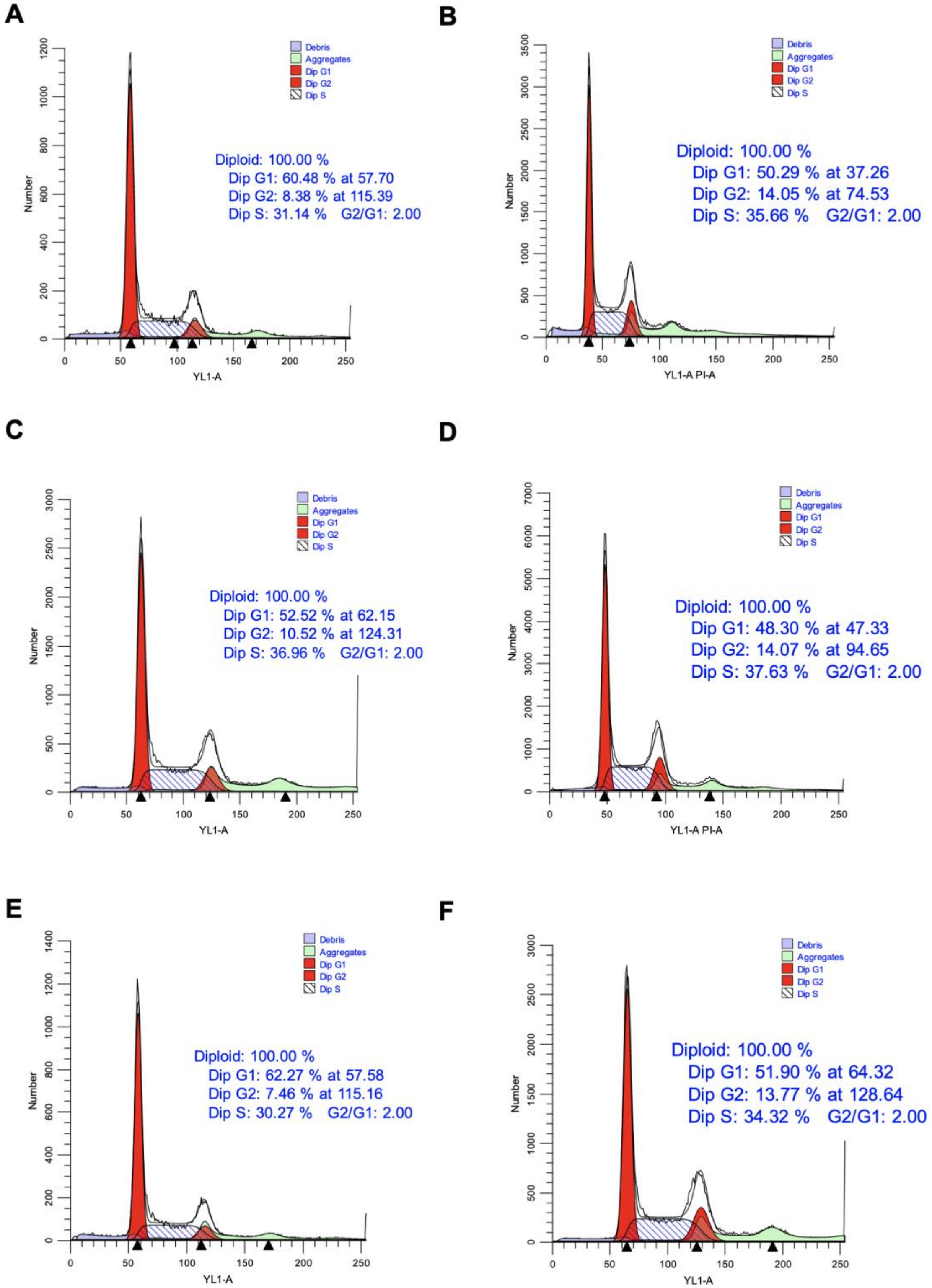

Figure S2. Representative histograms showing cell cycle profiles with siNC and siD12, illustrating the distribution across different cell cycle phases by flow cytometry. Cells were transfected with non-targeting control siRNA (siNC) **A, C, E,** or siRNA targeting DUSP12 (siD12) **B, D, F** for 72 h, stained with PI, and their cell cycle profile was analyzed by flow cytometry. Related to Figure S1B.

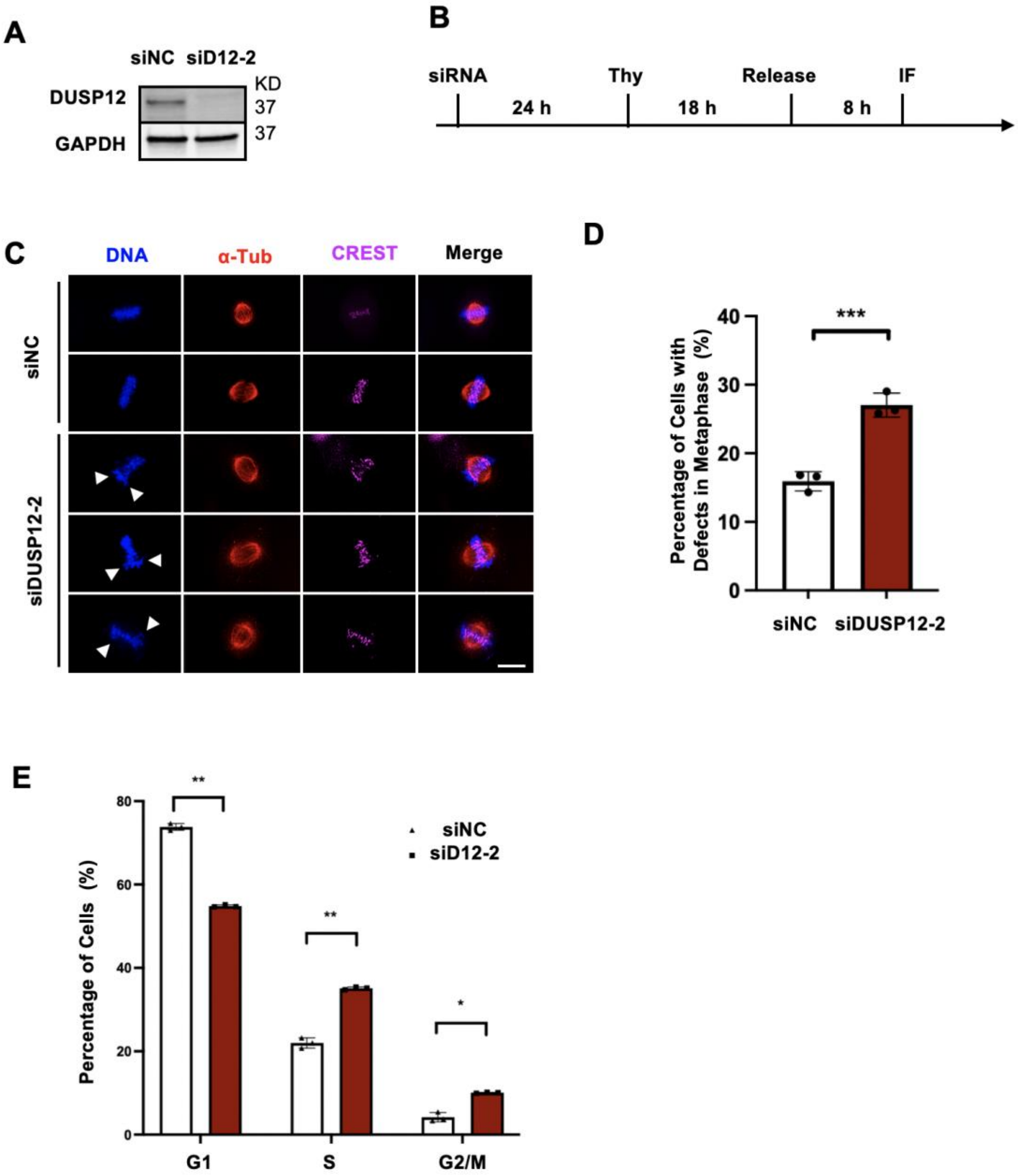

Figure S3. Knockdown of DUSP12 leads to cell division defects. A siRNA knockdown of endogenous DUSP12 in HeLa cells. Cells were transfected with negative control (siNC) or a second DUSP12 siRNA (siDUSP12-2) for 72 hours, followed by lysis and immunoblot analysis. **B** Schematic of experiments performed in (**C**). **C** Knockdown of DUSP12 leads to chromosome misalignment in metaphase. HeLa cells were treated with negative control siRNA or siDUSP12-2 before being fixed and co-stained with anti-CREST, DNA dye Hoechst 33342, and anti-α-tubulin antibodies. **D** Quantification of cells with misaligned chromosomes in metaphase (y-axis) for conditions shown in (**C**) (x-axis). **E** Flow cytometry cell cycle profiling of cells transfected with siNC or siDUSP12-2 for 72 h and stained with PI shows that DUSP12 depletion slows cells in G2/M phase. Data are shown as means ± SD. *p < 0.05, **p < 0.01, and ***p < 0.001 (unpaired two-tailed Student's t-test). Related to Figure 1.

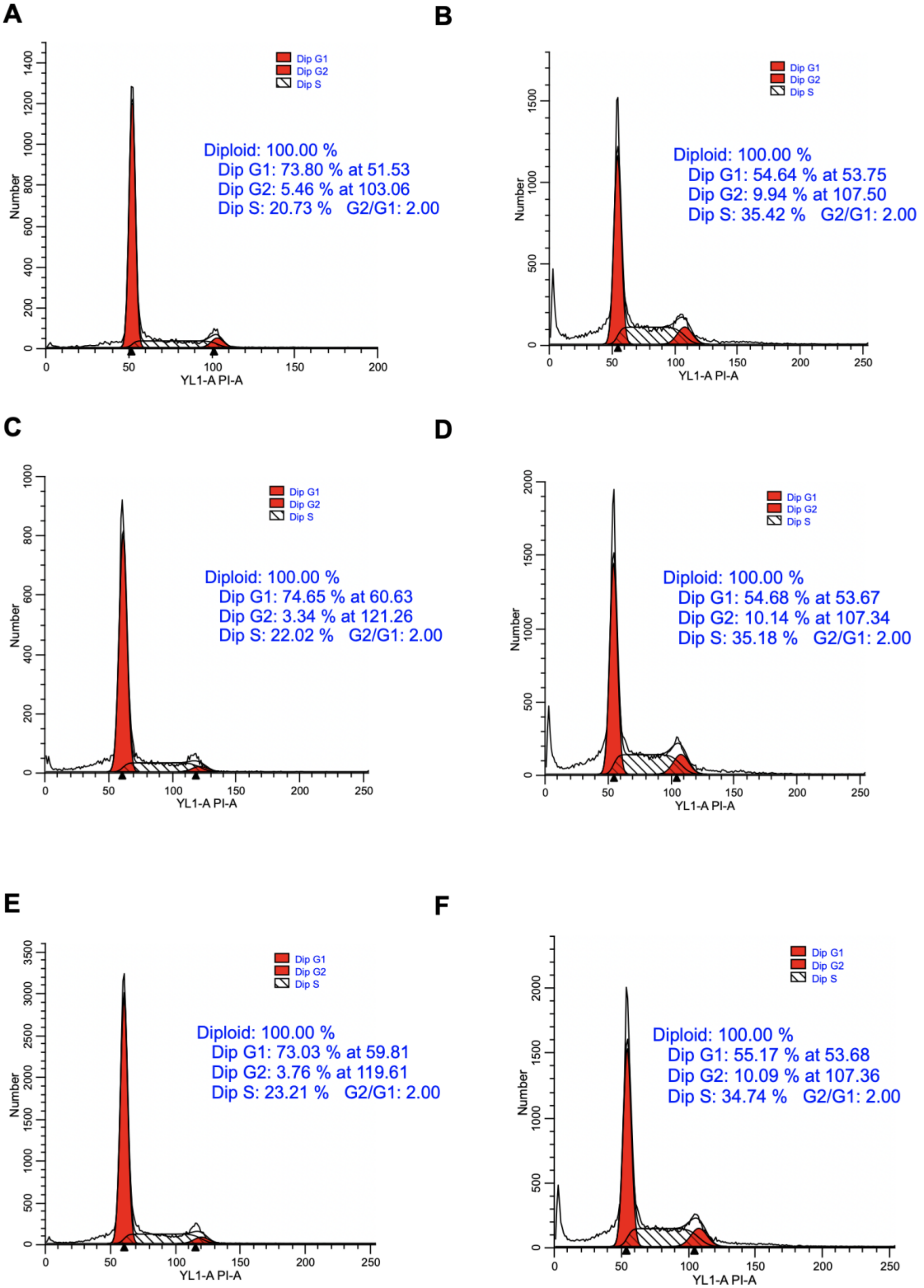

Figure S4. Representative histograms showing cell cycle profiles with siNC and siD12-2, illustrating the distribution across different cell cycle phases by flow cytometry. Cells were transfected with non-targeting control siRNA (siNC) **A, C, E,** or a second siRNA targeting DUSP12 **B, D, F** (siD12-2) for 72 h, stained with PI, and their cell cycle profile was analyzed by flow cytometry. Related to Figure S3E.

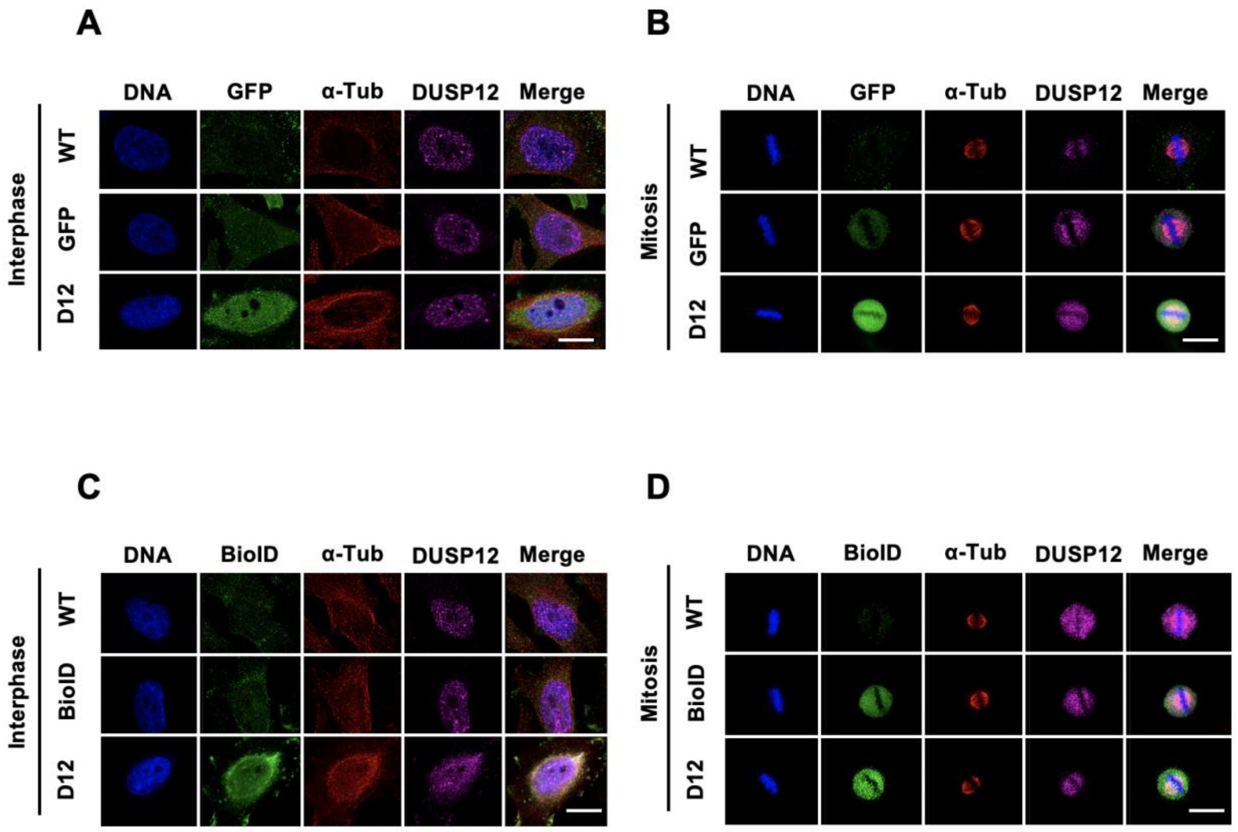

Figure S5. Endogenous DUSP12 co-localizes with GFP- and BioID-tagged DUSP12. A, B HeLa WT, LAP-GFP and LAP-DUSP12 HeLa stable cell lines were induced with Dox for 18 h before being fixed and co-stained with anti-GFP, anti-DUSP12, and anti-α-tubulin antibodies and the DNA dye Hoechst 33342. Representative images were captured in (**A**) interphase and (**B**) mitosis. **C, D** HeLa WT, BioID-only and BioID-DUSP12 stable cell lines were induced with Dox for 18 h before being fixed and co-stained with anti-BioID, anti-DUSP12, and anti-α-tubulin antibodies and the DNA dye Hoechst 33342. Representative images were captured in (**C**) interphase and (**D**) mitosis. Related to Figure 2.

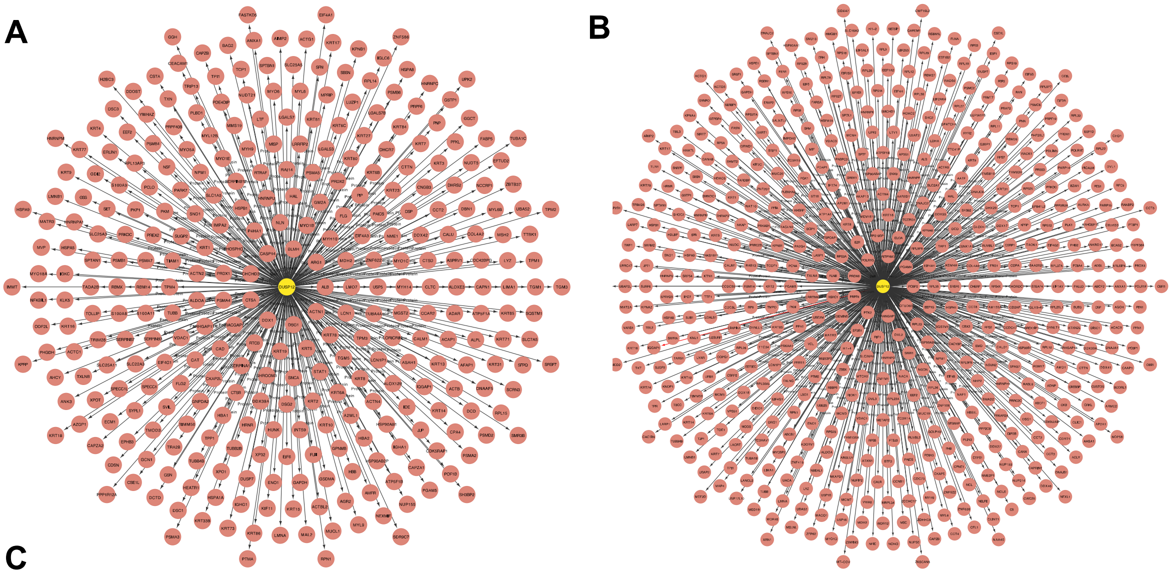

C

GO:ID	Ontology
GO:0000165	MAPK cascade
GO:0000186	activation of MAPKK activity
GO:0000187	activation of MAPK activity
GO:0000188	inactivation of MAPK activity
GO:0032872	regulation of stress-activated MAPK cascade
GO:0032873	negative regulation of stress-activated MAPK cascade
GO:0043409	negative regulation of MAPK cascade
GO:0043410	positive regulation of MAPK cascade
GO:0051403	stress-activated MAPK cascade
GO:1903753	negative regulation of p38MAPK cascade
GO:0032874	positive regulation of stress-activated MAPK cascade

Figure S6. Proteomic analysis DUSP12. A, Summary of the DUSP12 protein-protein interaction (PPI) network identified by mass spectrometry. **B**, Summary of the DUSP12 protein proximity (PP) network identified by mass spectrometry. **C**, Gene Ontology (GO) terms used to generate PPI and PP networks in Figure 2A and 2B. Related to Figure 2.

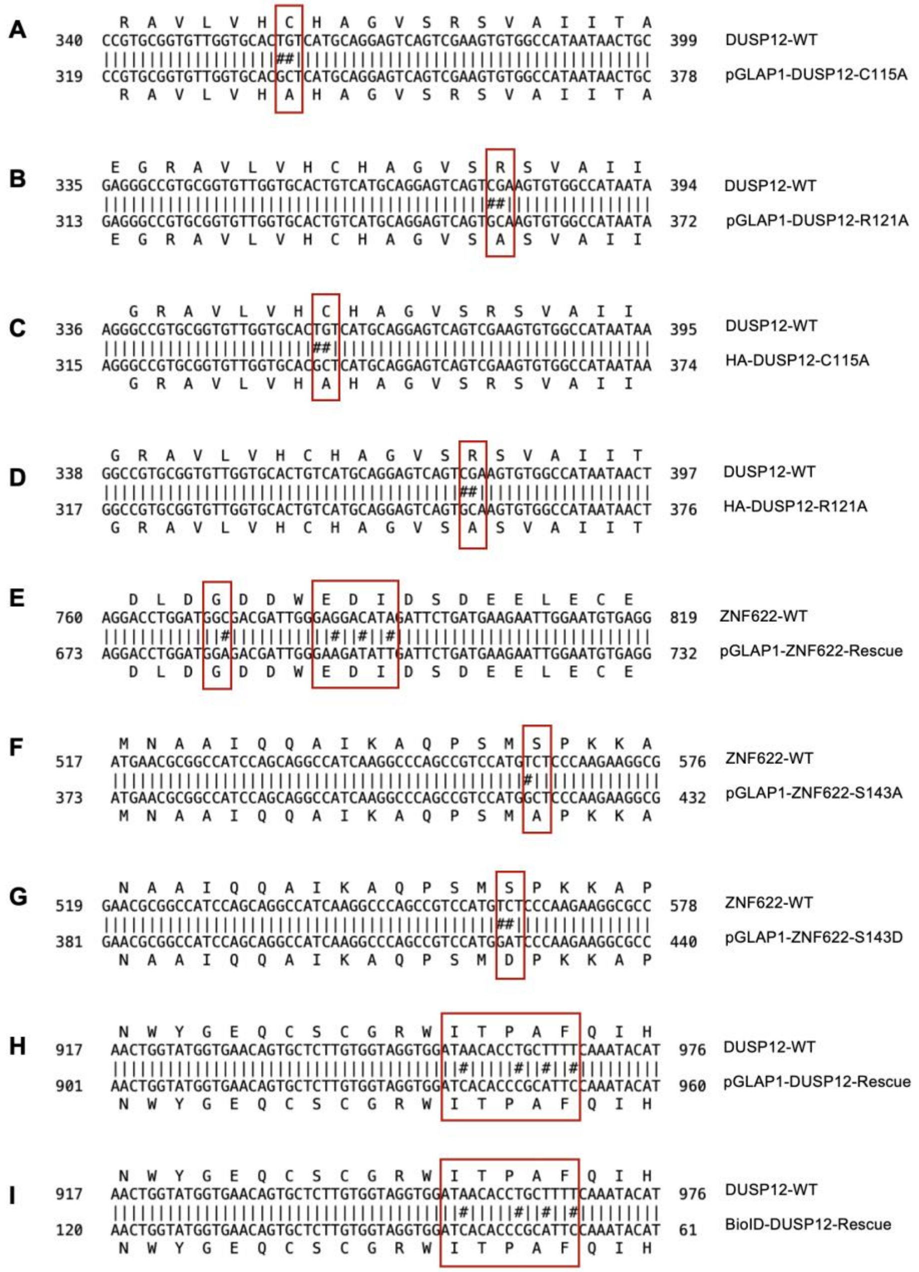

Figure S7. Verification of site-directed mutagenesis. **A** Sequencing data of pGLAP1-DUSP12-C115A showing successful mutation of DUSP12-C115 into alanine (outlined by red box). **B** Sequencing data of pGLAP1-DUSP12-R121A showing successful mutation of DUSP12-R121 into alanine (outlined by red box). **C** Sequencing data of HA-DUSP12-C115A showing successful mutation of DUSP12-C115 into alanine (outlined by red box). **D** Sequencing data of HA-DUSP12-R121A showing successful mutation of DUSP12-R121 into alanine (outlined by red box). **E** Sequencing data of pGLAP1- ZNF622-Rescue showing successful mutation of ZNF622-WT into siRNA resistant mutant (highlighted in red boxes). **F** Sequencing data of pGLAP1- ZNF622-S143A showing successful mutation of ZNF622-S143 into alanine (outlined by red box). **G** Sequencing data of pGLAP1- ZNF622-S143D showing successful mutation of ZNF622-S143 into aspartate (outlined by red box). **H** Sequencing data of pGLAP1-DUSP12-Rescue showing successful mutation of DUSP12-WT into siRNA resistant mutant (highlighted in red boxes). **I** Sequencing data of BioID-DUSP12-Rescue showing successful mutation of DUSP12-WT into siRNA resistant mutant (highlighted in red boxes). Related to Figure 2, 3, and S8.

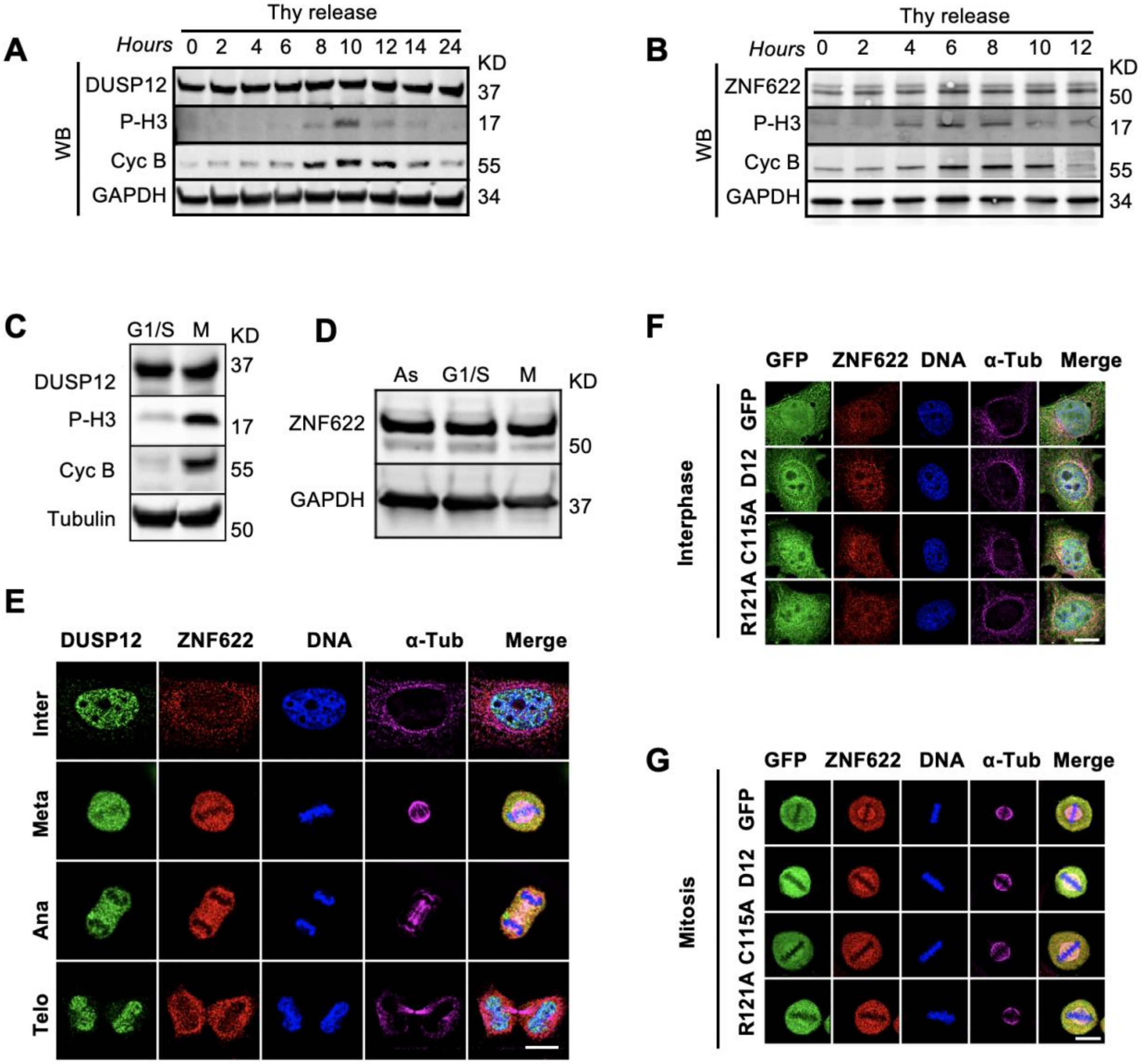

Figure S8. Correlation of expression between DUSP12 and ZNF622. A-D, DUSP12 and ZNF622 are expressed throughout cell cycle. HeLa cells were arrested in G1/S phase with 2 mM thymidine or in mitosis with 100 nM Taxol for 18 h. For (**A**) and (**B**), cells were released in to the cell cycle and harvested at the indicated time points. All cells were lysed and analyzed by immunoblot using the indicated antibodies. **E-G**, ZNF622 and DUSP12 share a similar endogenous localization pattern throughout the cell cycle. In (**E**) HeLa cells were fixed and co-stained with anti-DUSP12, anti-ZNF622 and anti-α-tubulin antibodies and the DNA dye Hoechst 33342. In (**F**) and (**G**) LAP-GFP, LAP-DUSP12, and LAP-DUSP12 catalytic mutant (C115A, R121A) HeLa stable cell lines were induced with Dox for 18 h then fixed and co-stained with anti-GFP, anti-ZNF622 and anti-α-tubulin antibodies and the DNA dye Hoechst 33342. Scale bar: 10 µm. Related to Figure 3.

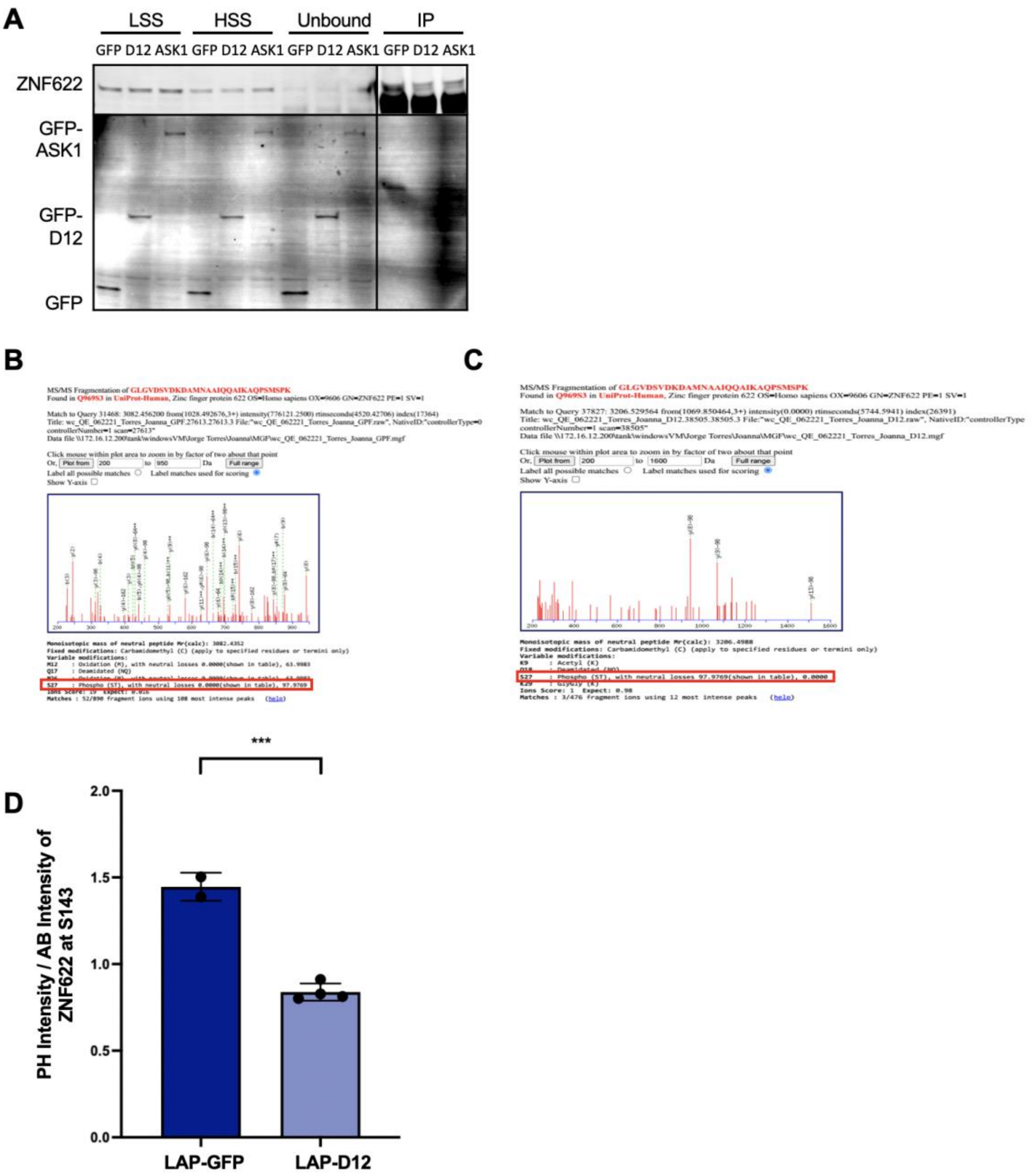

Figure S9. **Identification of ZNF622 phosphorylation site using mass spectrometry**. **A** LAP-GFP, LAP-ASK1, and LAP-DUSP12 HeLa stable cell lines were induced with Dox for 18 h before being harvested for pull downs with an anti-ZNF622 antibody for proteomics analysis shown in (**B**) and (**C**). **B** and **C** Mass Spectrometry-spectra corresponding to LAP-GFP and LAP-DUSP12 peptide fingerprints generated with trypsin digestion. **D** LAP-GFP and LAP-DUSP12 HeLa stable cell lines were induced with Dox for 18 h before being harvested for global phosphorylation analysis by mass spectrometry. Ratio of phosphorylation intensity to protein abundance of ZNF622 at S143 is shown in (**D**). Related to Figure 3.

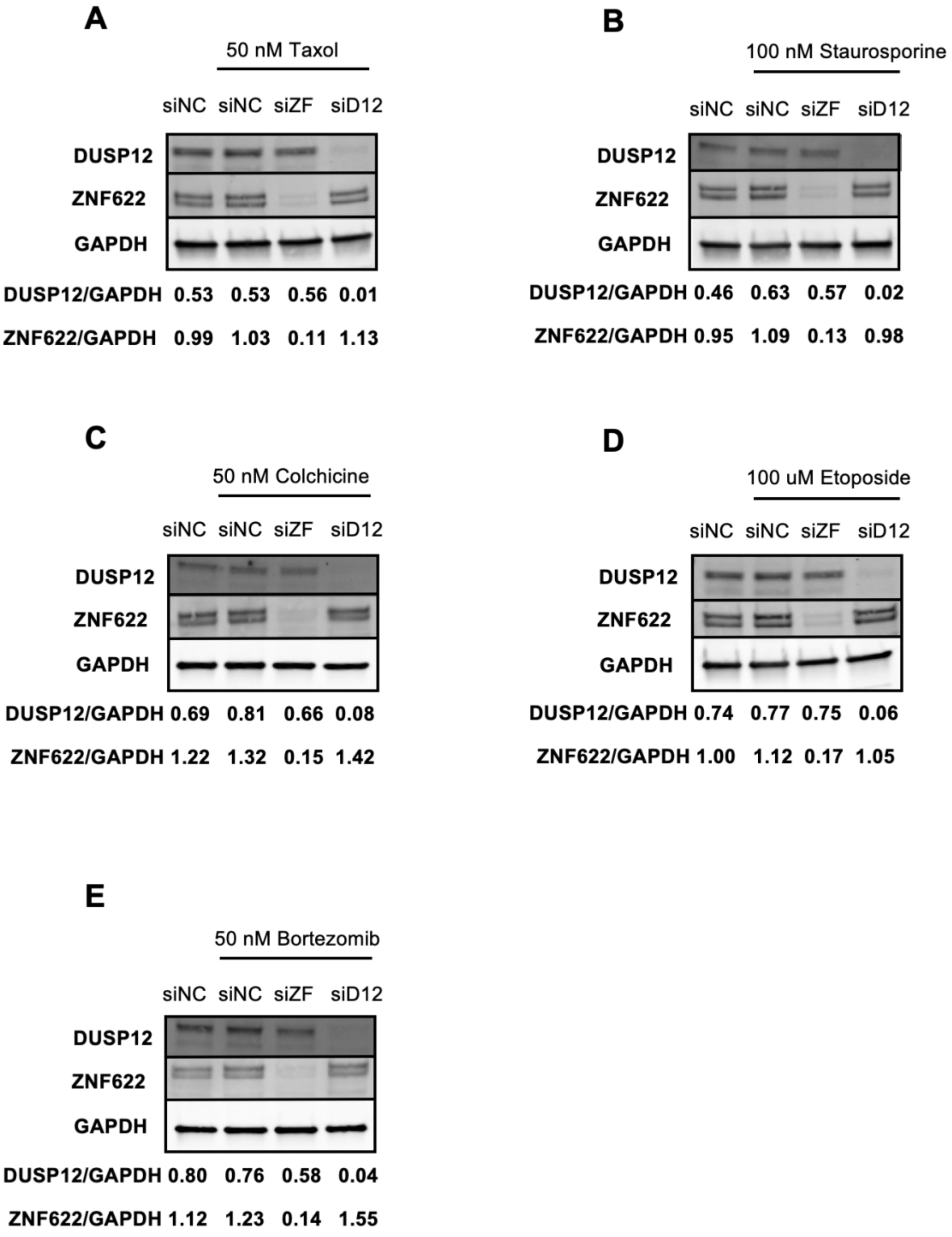

Figure S10. Validation of knockdown of DUSP12 and ZNF622 in response to stress induced apoptosis. HeLa cells were transfected with non-targeting control siRNA (siNC) or siRNA targeting ZNF622 (siZF) or DUSP12 (siD12) for 48 h, followed by treatment with 50 nM Taxol for 24 h (**A**), 100 nM Staurosporine for 24 h (**B**), 50 nM Colchicine for 24 h (**C**), 100 μM Etoposide for 24 h (**D**), or 50 nM Bortezomib for 24 h (**E**) before being lysed and analyzed by immunoblot. Ratios below the immunoblots indicate the normalized DUSP12 or ZNF622 levels. Related to Figure 4.

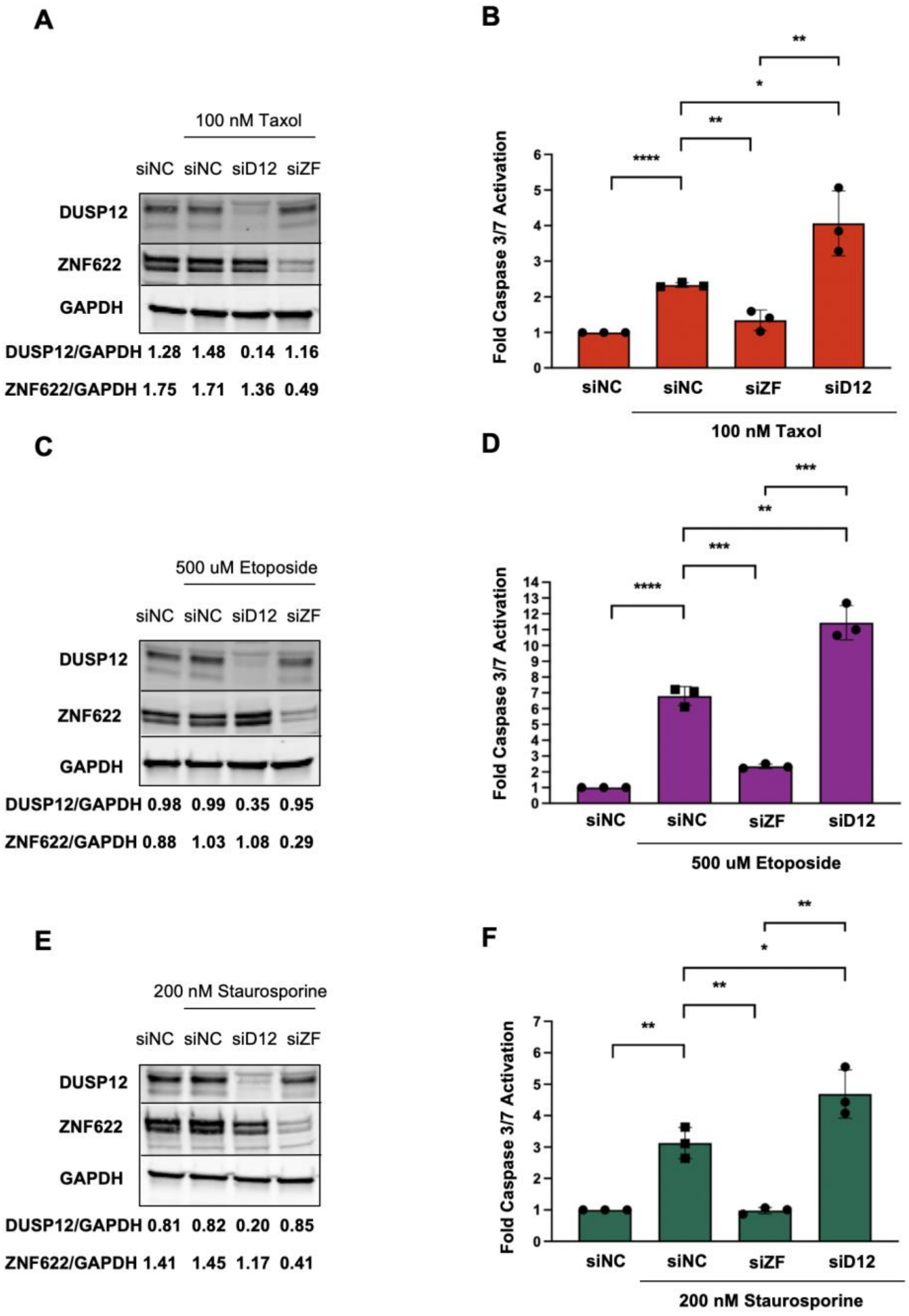

Figure S11. Knockdown of ZNF622 suppresses, while knockdown of DUSP12 promotes, stress induced apoptosis. **A, C, E** siRNA knockdown of endogenous DUSP12 or ZNF622 in HEPG2 cells. HEPG2 cells were reverse transfected with non-targeting control (siNC), ZNF622 (siZF) or DUSP12 (siD12) siRNA for 48 h, followed by treatment with 100 nM Taxol for 24 h (**A**), 500 µM Etoposide for 24 h (**B**), 100 nM Staurosporine for 24 h (**C**) before being lysed and analyzed by immunoblot. Ratios below the immunoblots indicate the normalized DUSP12 or ZNF622 levels. **B** Caspase 3/7 cleavage expressed as fold change relative to control (y-axis) for the conditions shown in (**A**). **D** Caspase 3/7 cleavage expressed as fold change relative to control (y-axis) for the conditions shown in (**C**). **F** Caspase 3/7 cleavage expressed as fold change relative to control (y-axis) for the conditions shown in (**E**). Data are shown as means ± SD. *p < 0.05, **p < 0.01, ***p < 0.001, and ****p < 0.0001 (unpaired two-tailed Student's t-test). Related to Figure 4.

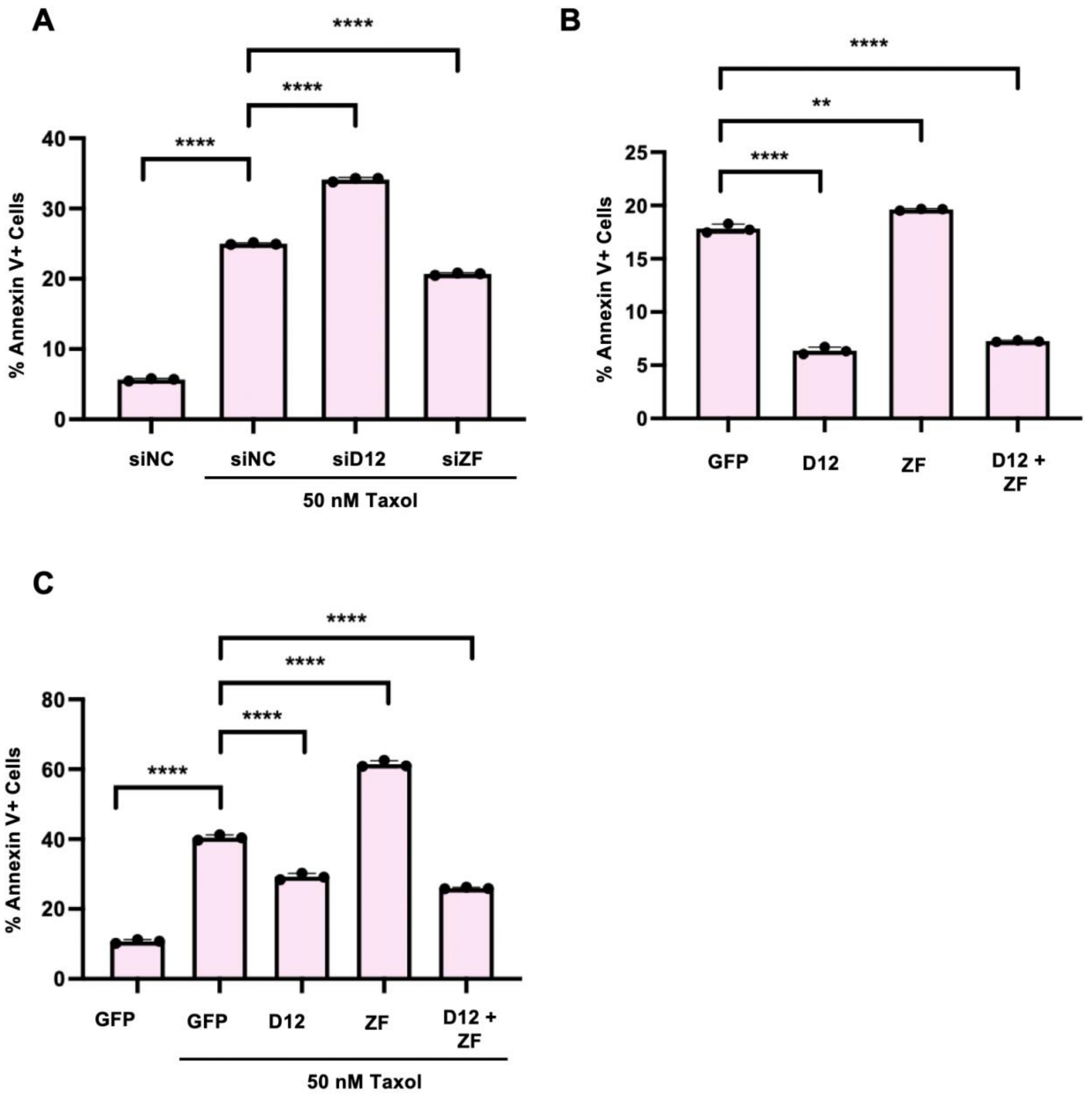

Figure S12. DUSP12 suppresses ZNF622 stress induced apoptosis. A HeLa cells were transfected with non-targeting control siRNA (siNC) or siRNA targeting ZNF622 (siZF) or DUSP12 (siD12) for 48 h, followed by treatment with 50 nM Taxol for 24 h. Quantification of total Annexin V+ cells (y-axis) for the indicated conditions (x-axis). **B** HeLa cells were transiently transfected with pGLAP1-GFP, pGLAP1-DUSP12, pGLAP1-ZNF622, or pGLAP1-DUSP12+pGLAP1-ZNF622 for 24 h. Quantification of total Annexin V+ cells (y-axis) for the indicated conditions (x-axis). **C** HeLa cells were transiently transfected with pGLAP1-GFP, pGLAP1-DUSP12, pGLAP1-ZNF622, or pGLAP1-DUSP12+pGLAP1-ZNF622 simultaneously with treatment with 50 nM Taxol for 24 h. Quantification of total Annexin V+ cells (y-axis) for the indicated conditions (x-axis). Cells were stained with Propidium Iodide to distinguish live and dead cells, and with Annexin V to distinguish non- and early- apoptotic cells. N=3 flow cytometric analyses of at least 10,000 cells. **$p < 0.01$ and ****$p < 0.0001$ (unpaired two-tailed Student's t-test). Related to Figure 4, 5, and 6.

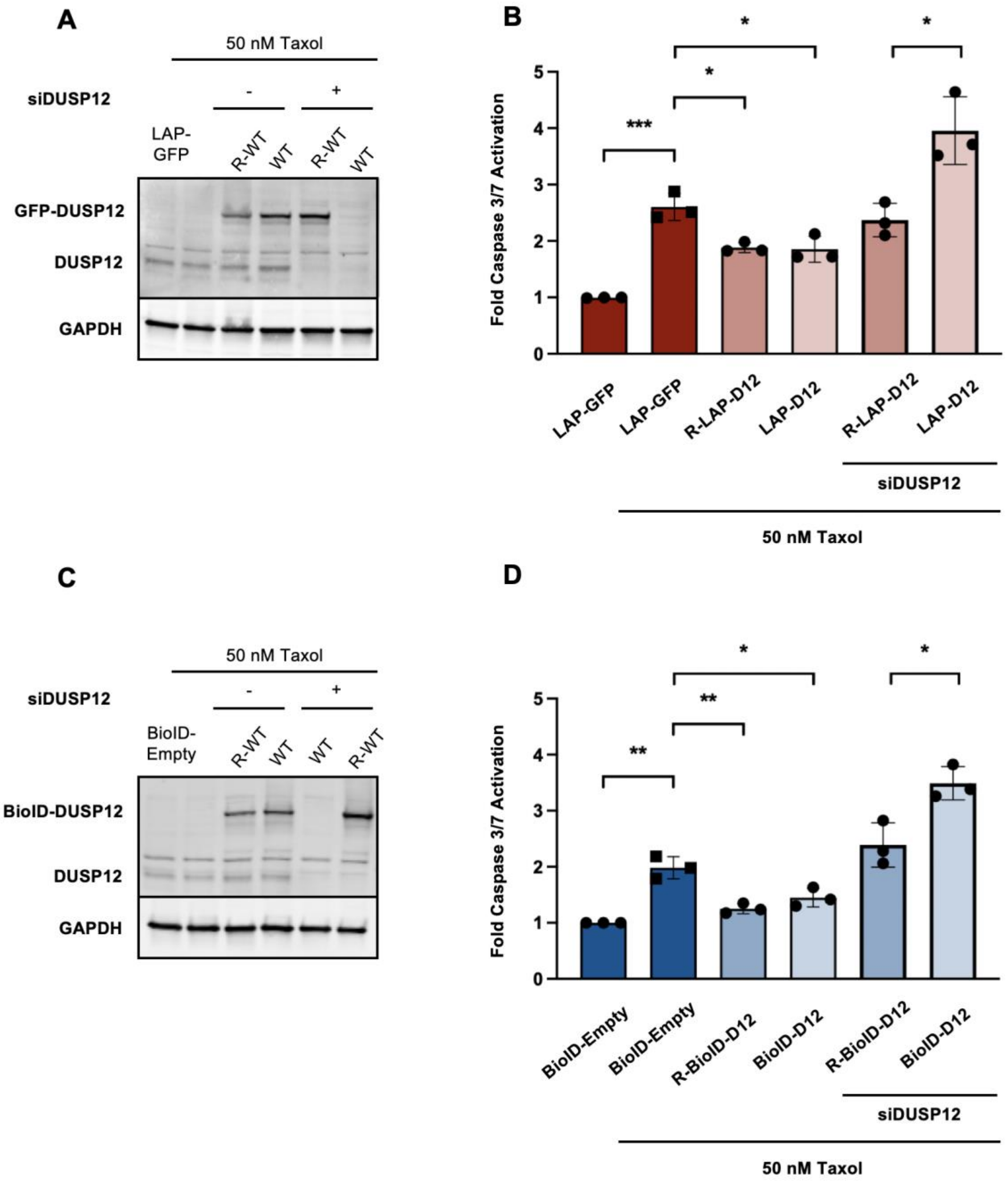

Figure S13. LAP-DUSP12 and BioID-DUSP12 suppress the siDUSP12 apoptotic phenotype. **A** LAP-DUSP12-WT and LAP-DUSP12-R-WT (resistant to siDUSP12, siRNA targeting DUSP12) HeLa inducible stable cell lines were transfected with siDUSP12 for 48 h, followed by treatment with 50 nM Taxol for 24 h, and induced with Dox during the last 16 h of the experiment to overexpress either GFP-DUSP12-WT or GFP-DUSP12-R-WT before being lysed and analyzed by immunoblot. **B** Caspase 3/7 cleavage expressed as fold change relative to control (y-axis) for the conditions shown in (**A**) (x-axis). **C** HeLa cells were transfected with siDUSP12 for 44 h followed by transfection with BioID-DUSP12-WT or BioID-DUSP12-R-WT for 26 h, and treated with 50 nM Taxol during the last 24 h of the experiment before being lysed and analyzed by immunoblot. **D** Caspase 3/7 cleavage expressed as fold change relative to control (y-axis) for the conditions shown in (**C**) (x-axis). Related to Figure 2 and 5.

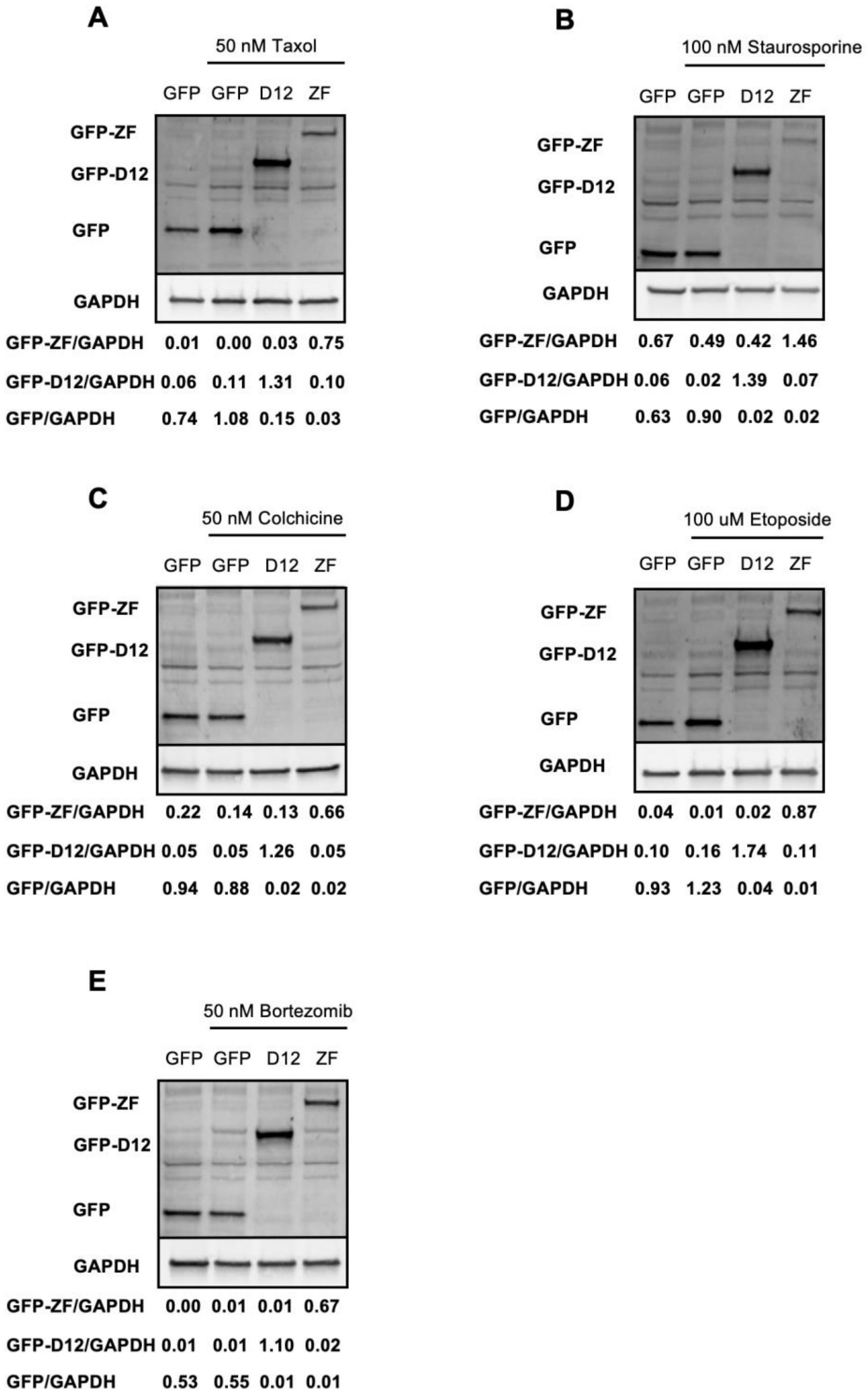

Figure S14. Validation of overexpression of DUSP12 and ZNF622 in response to stress induced apoptosis. LAP-GFP, LAP-DUSP12, and LAP-ZNF622 HeLa stable cell lines were induced with Dox and treated with 50 nM Taxol for 24 h (**A**), 100 nM Staurosporine for 24 h (**B**), 50 nM Colchicine for 24 h (**C**), 100 μM Etoposide for 24 h (**D**), or 50 nM Bortezomib for 24 h (**E**) before being lysed and analyzed by immunoblot. Ratios below the immunoblots indicate the normalized GFP, GFP-DUSP12 or GFP-ZNF622 levels. Related to Figure 5.

SUPPLEMENTARY MOVIE LEGENDS

Movie S1. Live-cell time-lapse microscopy movie of a representative siNon-targeting control siRNA treated HeLa-3xNLS-EBFP2 cell undergoing cell division. Related to Figure 1. Cells were transfected with control siRNA, treated as described in Figure 1E and imaged live one-hour post thymidine release for 17 h with an ImageXpress XL imaging system using a 20x air objective. Images were captured every five minutes with the DAPI channel and converted to AVI movies with ImageJ at one frame per second. Each frame represents a five-minute interval.

Movie S2. Live-cell time-lapse microscopy movie of representative siDUSP12 treated HeLa-3xNLS-EBFP2 cell undergoing cell division. Related to Figure 1. Cells were transfected with DUSP12 siRNA, treated as described in Figure 1E, and imaged live one-hour post thymidine release for 17 h with an ImageXpress XL imaging system using a 20x air objective. Images were captured every five minutes with the DAPI channel and converted to AVI movies with ImageJ at one frame per second. Each frame represents a five-minute interval.

SUPPLEMENTARY TABLE LEGENDS

Table S1. List of Gene Ontology (GO) annotations identified in the GO enrichment analysis of the DUSP12 interacting protein network and protein proximity association network analyses. Includes GO terms identified in the GO enrichment analysis with Biological Process category, GO term IDs, GO annotations, and the frequency of the GO terms identified in the GO enrichment analysis.

Table S2. List of key reagents and resources used in this study.

Table S3. List of all protein identifications from LAP purifications, including protein accession number, number of distinct peptides assigned for each protein, and derived protein identification probability.

Table S4. List of all protein identifications from BioID2 purifications, including protein accession number, number of distinct peptides assigned for each protein, and derived protein identification probability.

SUPPLEMENTARY METHODS

EXTENDED GLOBAL PHOSPHOPROTEOMIC ANALYSIS PROTOCOL

Mass spectrometry proteomics acquisition

Samples were lysed in 8 M Urea (Promega, Madison WI) in LoBind tubes (Eppendorf, Hamburg Germany). Lysed samples were sonicated using a probe sonicator 2x for 10 seconds On and 10 seconds Off at 10% amplitude, and protein was quantified using a Bradford assay. Approximately 200 µg of protein for each sample was used for further processing, starting with reduction using a 10 mM final concentration of tris-(2-carboxyethyl) (TCEP), followed by 40 mM final concentration of chloroacetamide both for 30 minutes at 23 °C with shaking at 1100 rpm. Before protein digestion, the 8 M Urea was diluted 8-fold with 100 mM Tris-HCl (pH 8) to permit the activity of the proteolytic enzyme trypsin. Trypsin (Promega) and Lys-C (Wako) was added at a 1:100 (wt/wt) enzyme-substrate ratio and placed in a thermomixer at 23 °C overnight (16 h) with shaking at

1000 rpm. Following digestion, 10% trifluoroacetic acid (TFA) was added to each sample to a final pH of approximately 2-3. Samples were then desalted using a vacuum manifold with 30 mg HLB 1cc vacuum cartridges (Waters). Each cartridge was activated with 1 mL of 80% acetonitrile (ACN)/0.1% TFA, then equilibrated with 3 x 1 mL of 0.1% TFA. Samples were load through the cartridges twice, then washed with 3 x 1 mL of 0.1% TFA, and finally samples were eluted with 1 x 0.8 mL 50%ACN/0.25% formic acid (FA). Approximately 10% of the desalted peptides were separated for global proteomics analysis and the remaining 90% were used for phosphopeptide enrichment. Both the fractions were then dried by vacuum centrifugation in a SpeedVac (Labconco, Kansas City MO). For phosphopeptide enrichment, Ti-IMAC beads (Resyn Biosciences, Pretoria South Africa) were aliquoted in a 1:10 (w/w) peptide:beads ratio and equilibrated three times with binding buffer (0.1 M glycolic acid in 80% acetonitrile (ACN), 5% TFA). Dried peptide samples were resuspended in 200 µL of binding buffer, added to the equilibrated beads, and incubated for 30 min at 23 °C, 1500 rpm. The unbound fraction was discarded, and beads were washed sequentially once each with 200 µL of the binding buffer, wash buffer 1 (60% ACN, 1% TFA, 200 mM NaCl), wash buffer 2 (60% ACN, 1% TFA), and finally with LC-MS grade water. Enriched phosphopeptides were eluted twice by incubating the beads with 150 µL of 1% (v/v) ammonium hydroxide (Sigma, St. Louis MO) in LC-MS grade water (Fisher Scientific, Waltham MA) for 10 min at 23 °C, 1500 rpm. The eluted peptides were transferred to a new protein LoBind tube containing 50 µL of 10% (v/v) formic acid in LC-MS grade water. Both eluates were pooled and an addition 50 µL of 10% (v/v) formic acid was added before enriched samples were dried and resuspended in 0.1% formic acid, and a volume corresponding to 500 ng of peptides was analyzed using a timsTOF HT mass spectrometer (Bruker Daltonics, Billerica MA).

Mass spectrometry proteomics acquisition

Dried peptides were resuspended in 0.1% (v/v) FA in MS grade water (Fisher Scientific) and analyzed on a timsTOF HT mass spectrometer, paired with a Vanquish Neo UHPLC system. Mobile phase A consisted of 0.1% (v/v) FA in MS grade water (Fisher Scientific), and mobile phase B consisted of 0.1% (v/v) FA in 100% MS grade Acetonitrile (Fisher Scientific). The LC was operated in trap-and-elute mode, where the peptides were first trapped onto a PepMap Neo Trap column (5 mm, 100 Å pore size, 5 µm particle size) and then reversed-phase separated using gradients mentioned below on an Aurora Elite C18 reverse phase column (15 cm, 100 Å pore size, 1.5 µm particle size for captive spray, IonOptiks), kept at 50 °C using a column oven for Bruker Captive Spray source (Sonation Lab Solutions, Biberach Germany), and ionized in a CaptiveSpray source (Bruker Daltonics) at 1700 V. For global proteome analysis, the %B gradient used was: 5% to 35% over 37 min at 0.3 µL/min, then to 45% in the next 4 mins, and 60% in the next 1 min, followed by an increase to 95% B over 3 min. For phosphoproteome analysis, the %B gradient used was 3% to 16% over 26 min, 30% in the next 11.5 min, 45% in the next 4 min, and 60% B over 1 min at 0.3 µL/min flow rate, followed by an increase to 95% B over 3 min. All MS data was acquired in dia-PASEF mode with variable isolation window widths in the m/z vs ion mobility plane. These windows were adjusted to maximize the coverage of precursor ions. For abundance proteomics, MS1 scans were acquired from 100–1700 m/z and a dual-TIMS analyzer ramp rate of 9.42 Hz, with 100 ms accumulation and ramp times (100% duty cycle).The ion mobility range was 0.60–1.60 V·s/cm², isolation windows were 24 Da (1 Da overlap) with 44 mass steps per 1.27 s cycle, and collision energy decreased linearly from 59 eV at $1/K_0$ = 1.41 V·s/cm² to 20 eV at $1/K_0$ = 0.71 V·s/cm², collecting MS/MS spectra from 315.5–1328.5 m/z. For phosphoproteomics, MS1 scans were acquired from 100–1700 m/z and a dual-TIMS analyzer ramp rate of 9.42 Hz, with 100 ms accumulation and ramp times (100% duty cycle). The ion mobility range was 0.60–1.60 V·s/cm², isolation windows were 25 Da (1 Da overlap) with 44 mass steps per 1.27 s cycle, and collision energy decreased linearly from 59 eV at $1/K_0$ = 1.41 V·s/cm² to 20 eV at $1/K_0$ = 0.71 V·s/cm², collecting MS/MS spectra from 330.8–1387.8 m/z.

Mass spectrometry proteomics data search and quantitative analysis

The raw files were processed with Spectronaut (Biognosys, Zurich Switzerland) with the directDIA+ (Deep) search algorithm, its in silico-derived DIA analysis. Carbamidomethylation (cysteine) was set as a fixed modification for database search. Acetylation (protein N-term), oxidation (methionine), and phosphorylation (serine, threonine, tyrosine; only for phosphoproteomics dataset) were set as variable modifications. Reviewed human protein sequences (downloaded from UniProt, October 6, 2023) were used for spectral matching. The false discovery rates for the PSM, peptide, and protein groups were set to 0.01, and the minimum localization threshold for PTM was set to zero. For MS2-level area-based quantification, the cross-run normalization option was unchecked (normalization was performed later using MSstats), and the probability cutoff was set to zero for the PTM localization. Quantitative analysis was performed in the R statistical programming language (v.4.5.1). Initial quality control analyses, including inter-run clustering, correlations, principal component analysis (PCA), peptide and protein counts, and intensities were completed in R. Statistical analysis of phosphorylation and protein abundance changes between exposed and control samples were computed using the R package MSstats (v.4.16.1). For protein abundance, all peptides mapping to the same proteins were summarized together using the Tukey's Median Polish approach. For phosphoproteomics, all peptides containing the same set of phosphorylated sites were summarized together into phosphorylation site groups using Tukey's Median Polish. For both phosphopeptide and protein abundance MSstats pipelines, MSstats performs normalization by median equalization, imputation was turned Off (set to FALSE), and statistical tests of differences in intensity between conditions was calculated using default settings in MSstats. Specifically, MSstats calculates log2 fold changes as the ratio of averaged (across replicates) protein or phosphopeptide intensities between conditions, uses a Student's t-test for p-value calculation and the Benjamini-Hochberg method of FDR estimation to adjust p-values.
