# Supplementary material for: DUSP12 promotes cell cycle progression and protects cells from ZNF622 mediated apoptosis
Source: Cell Death Dis. 2026 Mar 18;17(1):315. doi: 10.1038/s41419-026-08618-z (PMC13039736; doi:10.1038/s41419-026-08618-z)
Supplement: Supplementary file 2 — Full Uncropped Western Blots [file 41419_2026_8618_MOESM2_ESM.pdf]

Fig. 1A

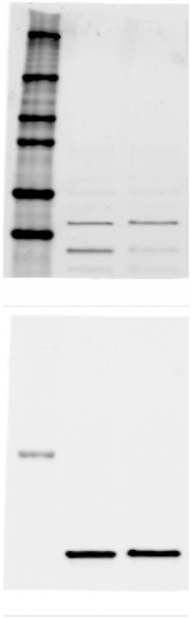

DUSP12

GAPDH

Fig. 2C

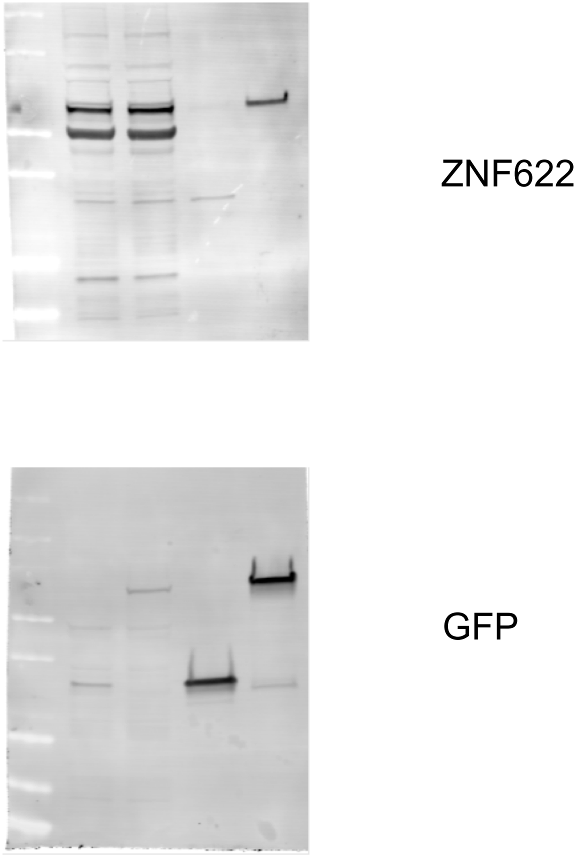

Fig. 2D

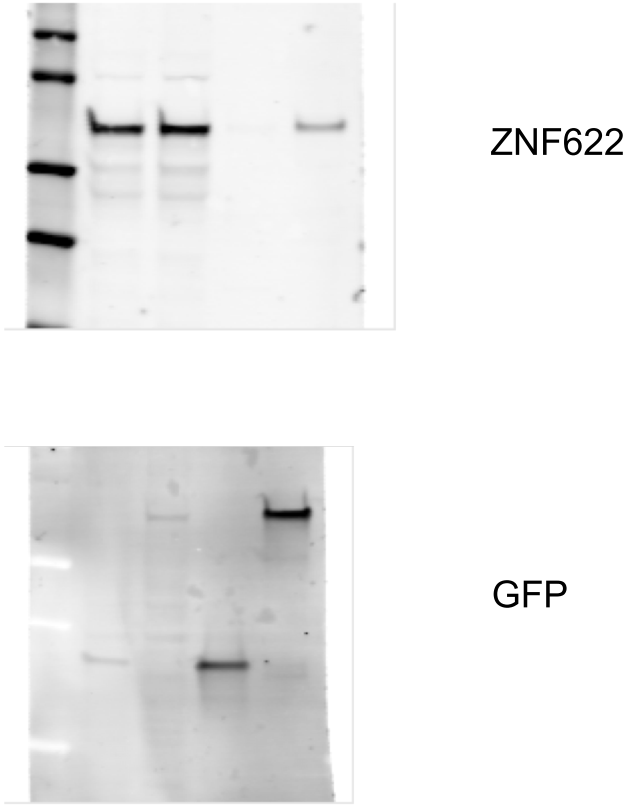

Fig. 2F

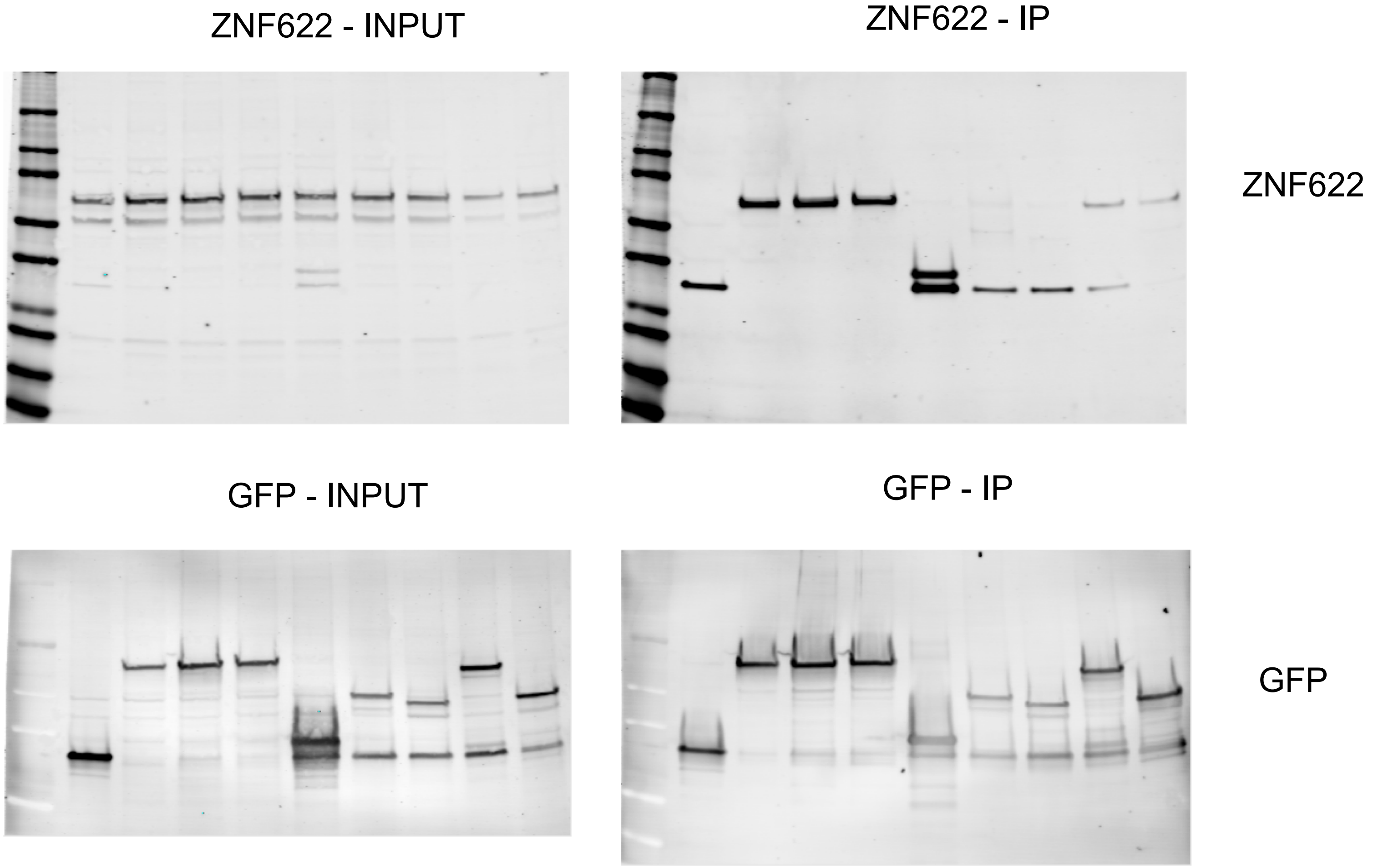

Fig. 2G

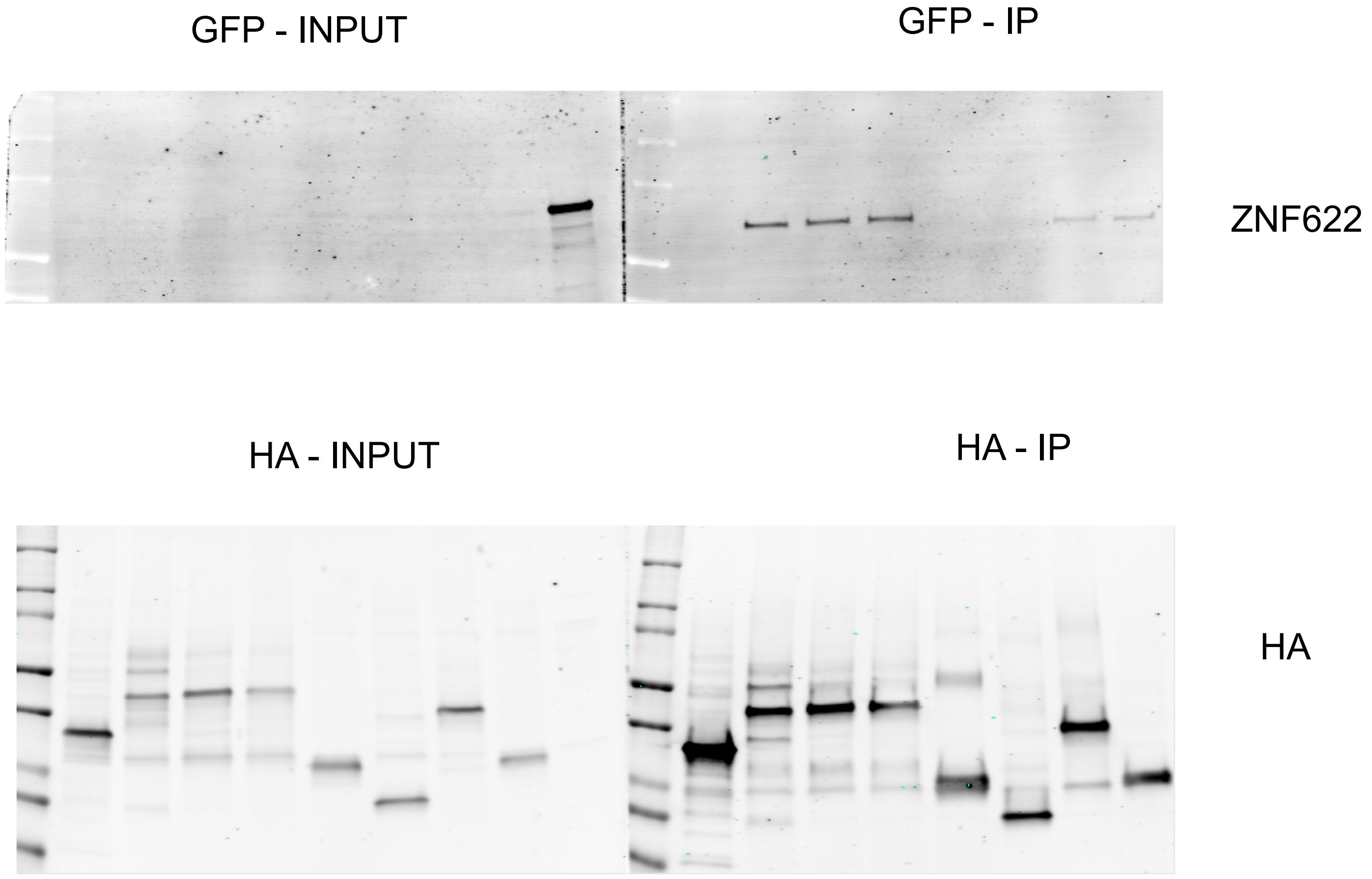

Fig. 3A

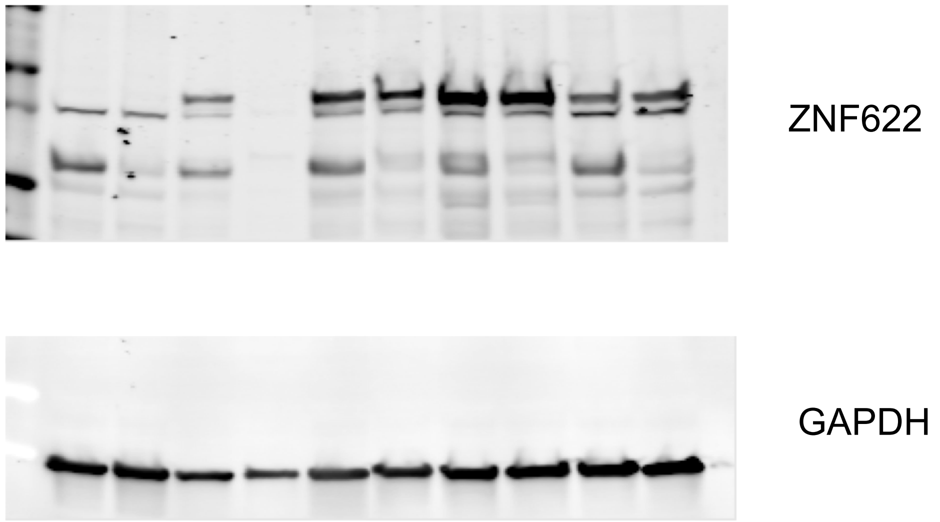

Fig. 6A

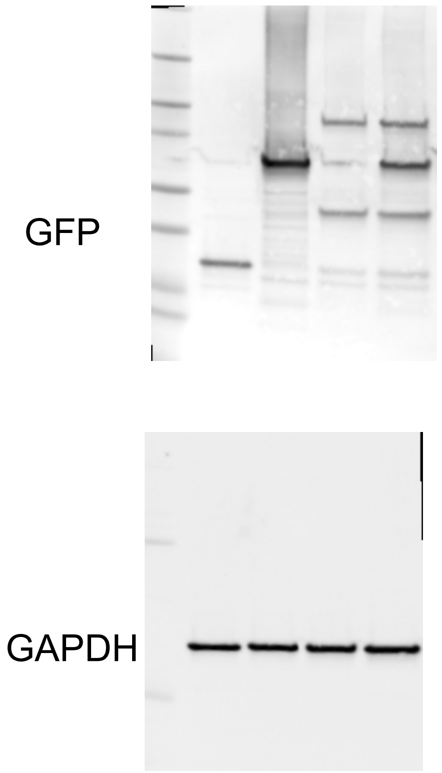

Fig. 6C

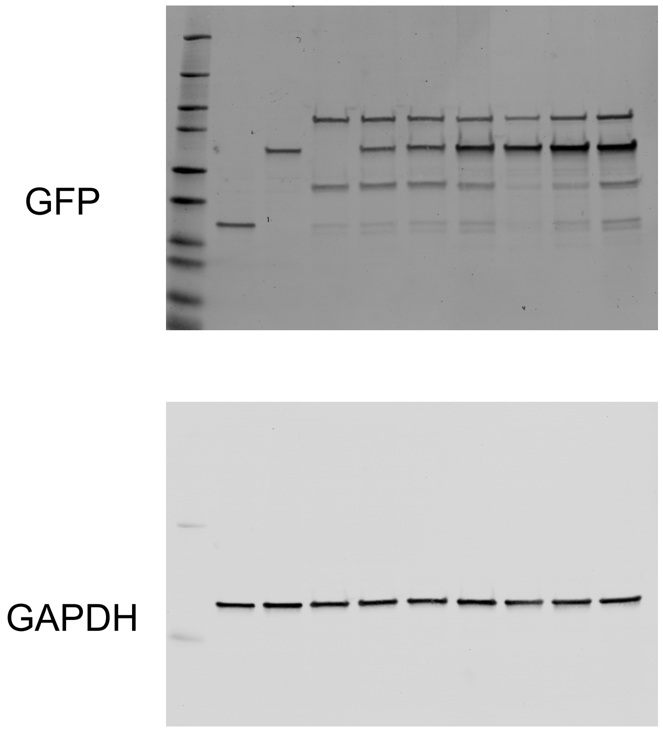

Fig. 7A

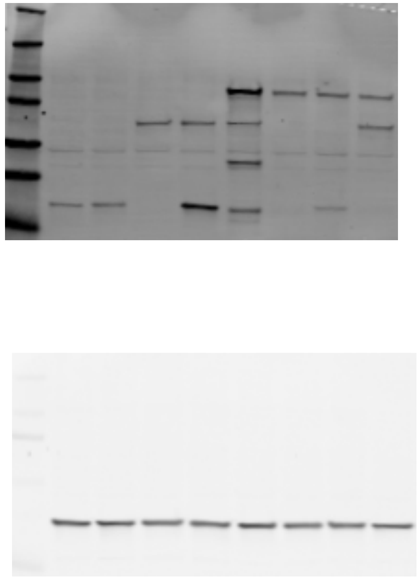

GFP

GAPDH

Fig. S1A

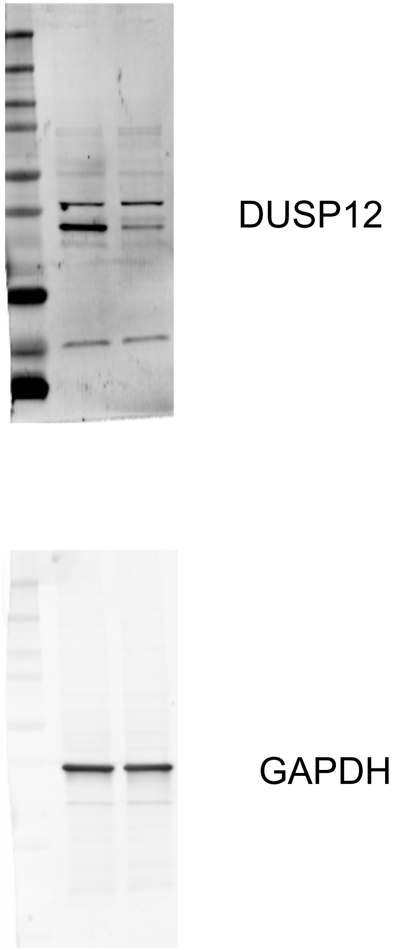

Fig. S1C

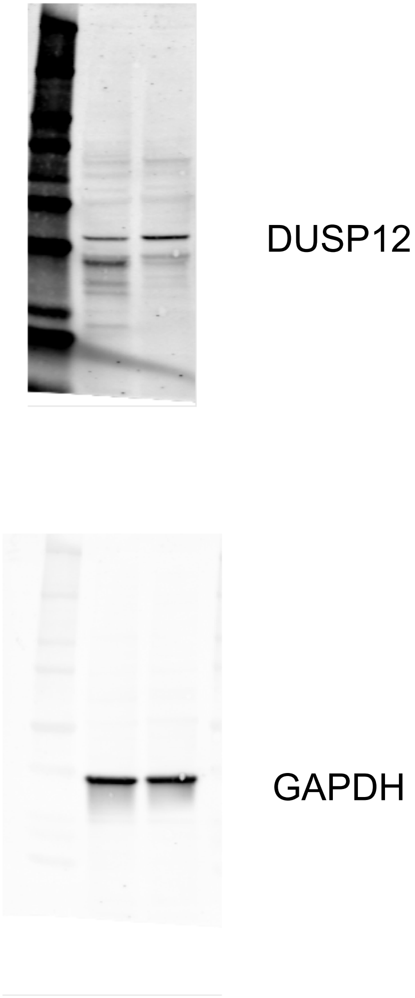

Fig. S3A

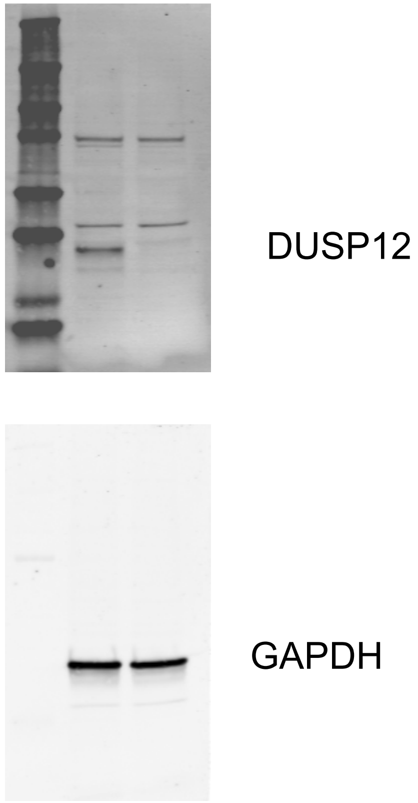

Fig. S8A

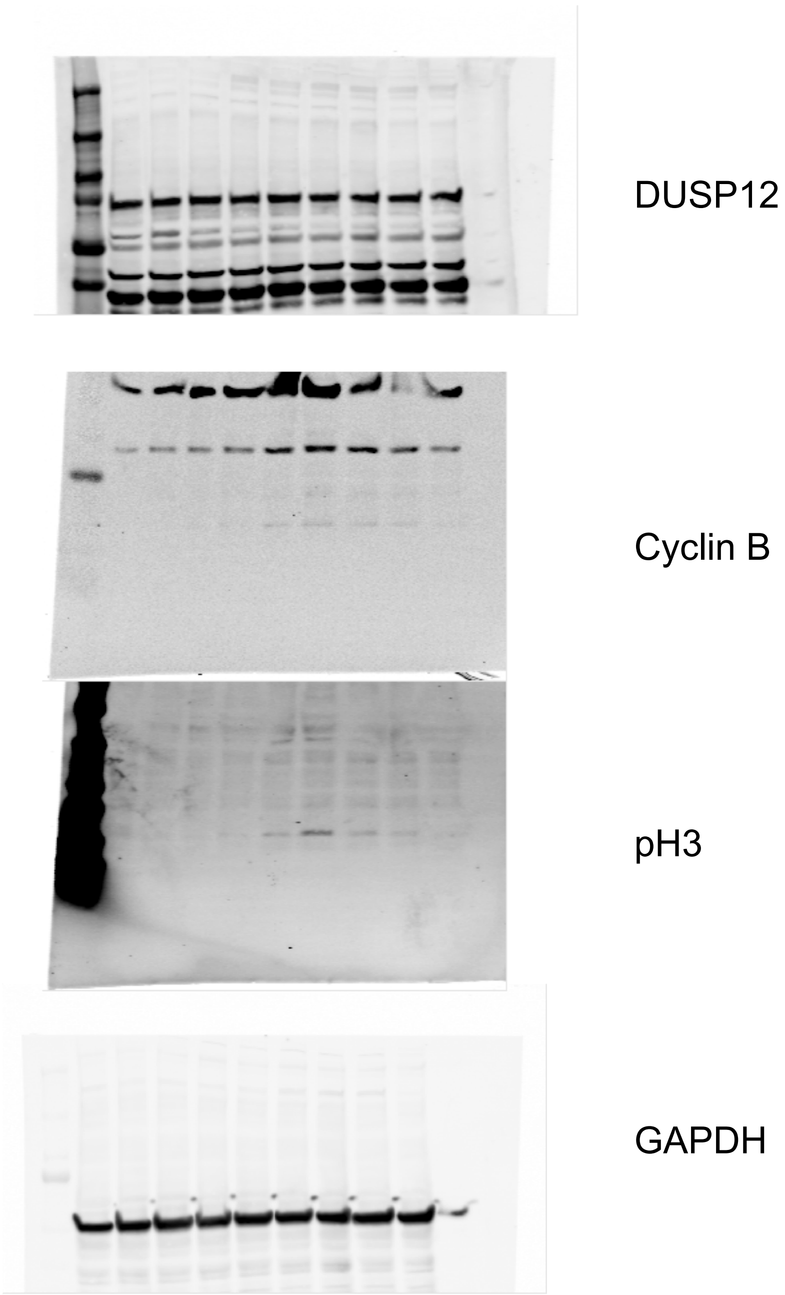

Fig. S8B

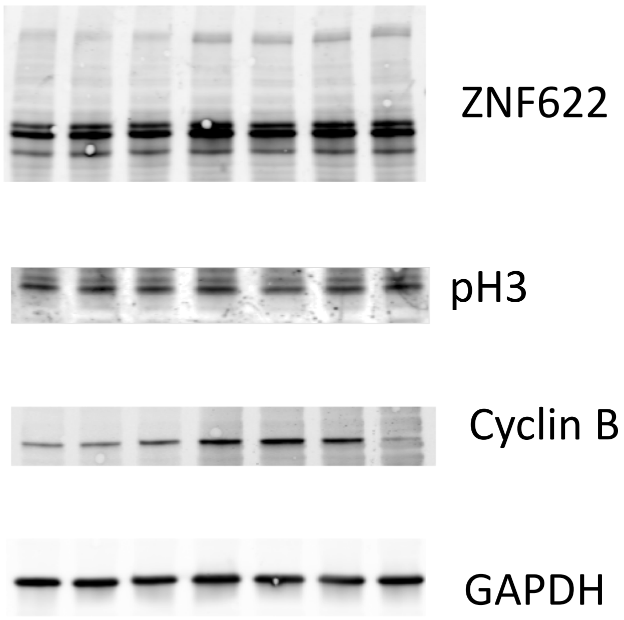

Fig. S8C

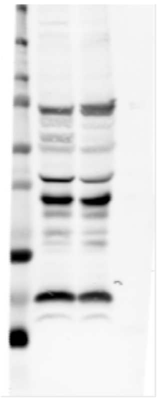

DUSP12

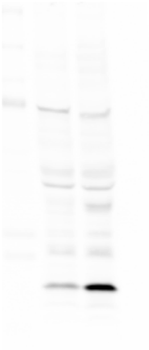

pH3

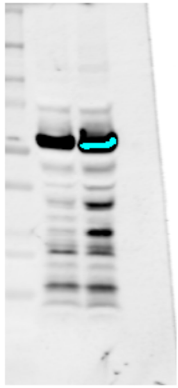

Cyc B

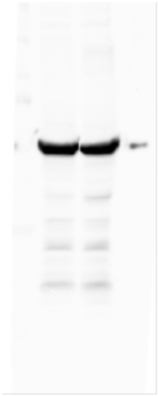

Tub

Fig. S8D

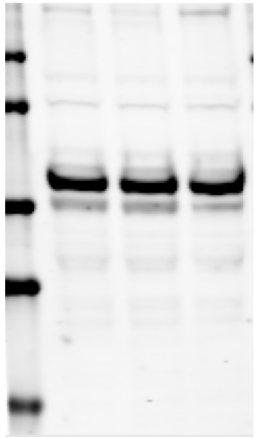

ZNF622

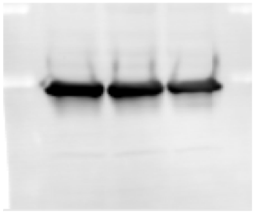

GAPDH

Fig. S9A

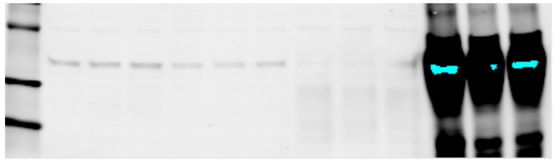

ZNF622 dark exposure

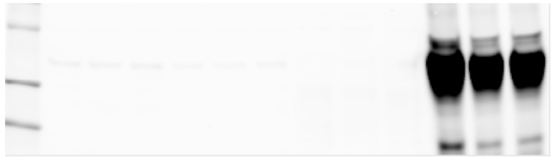

ZNF622 light exposure

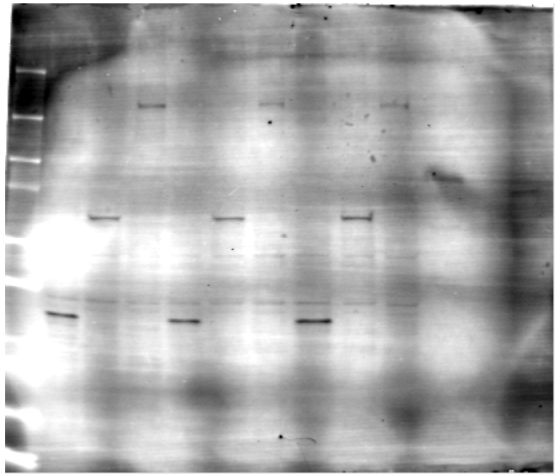

GFP

Fig. S10A

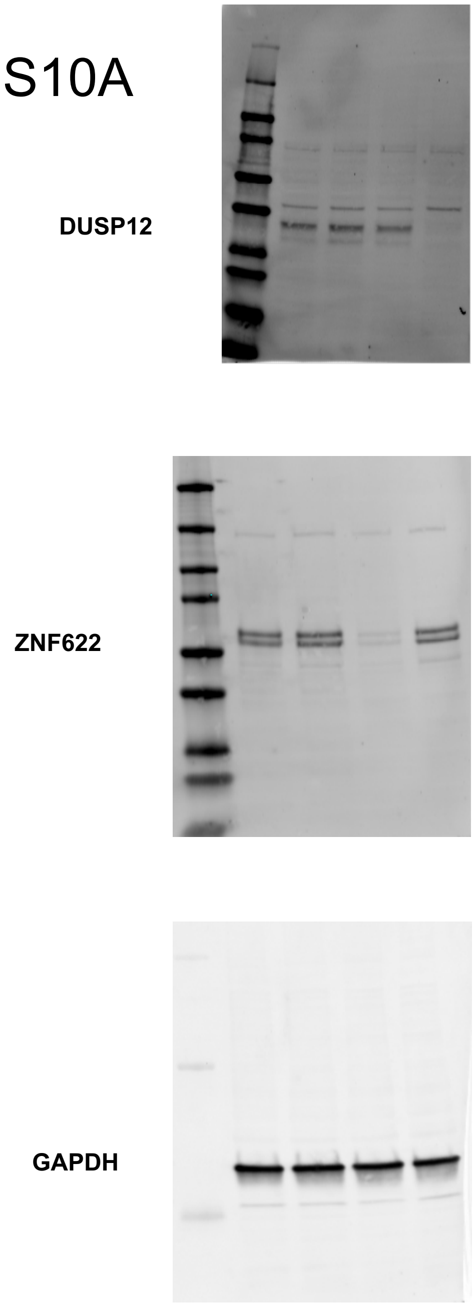

Taxol

Fig. S10B

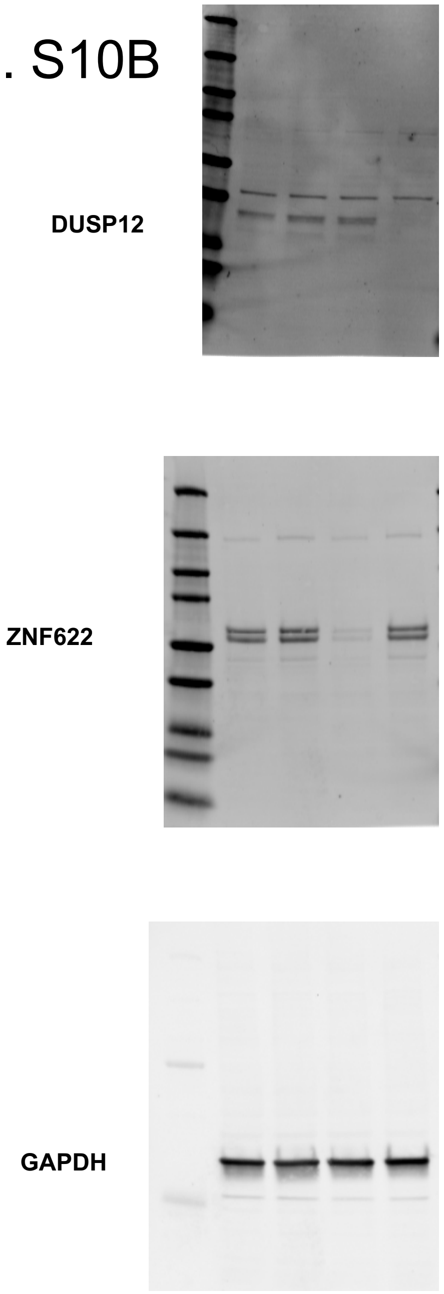

Staurosporine

Fig. S10C

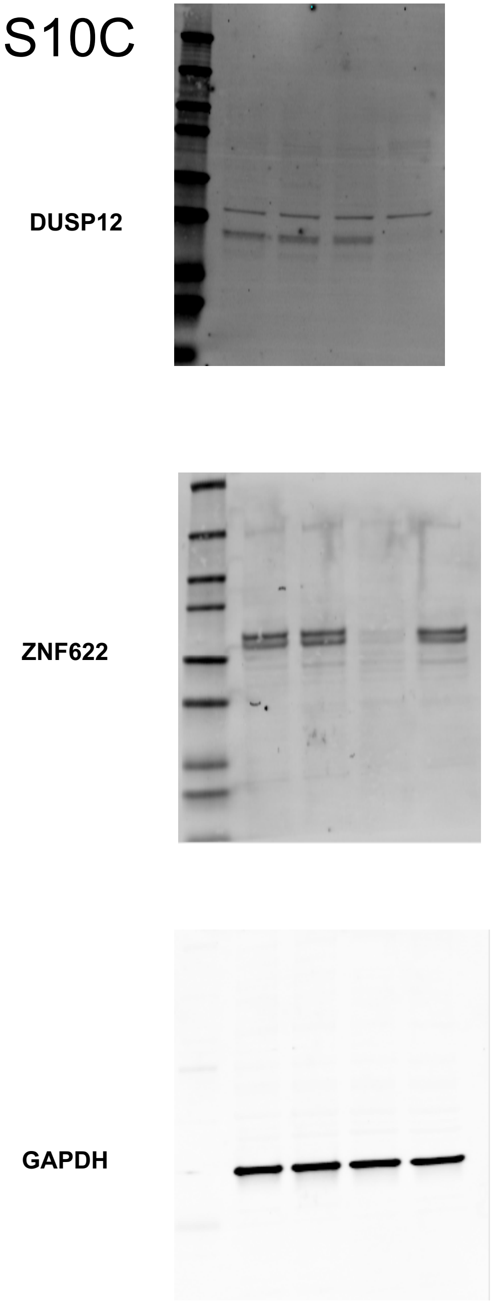

Colchicine

Fig. S10D

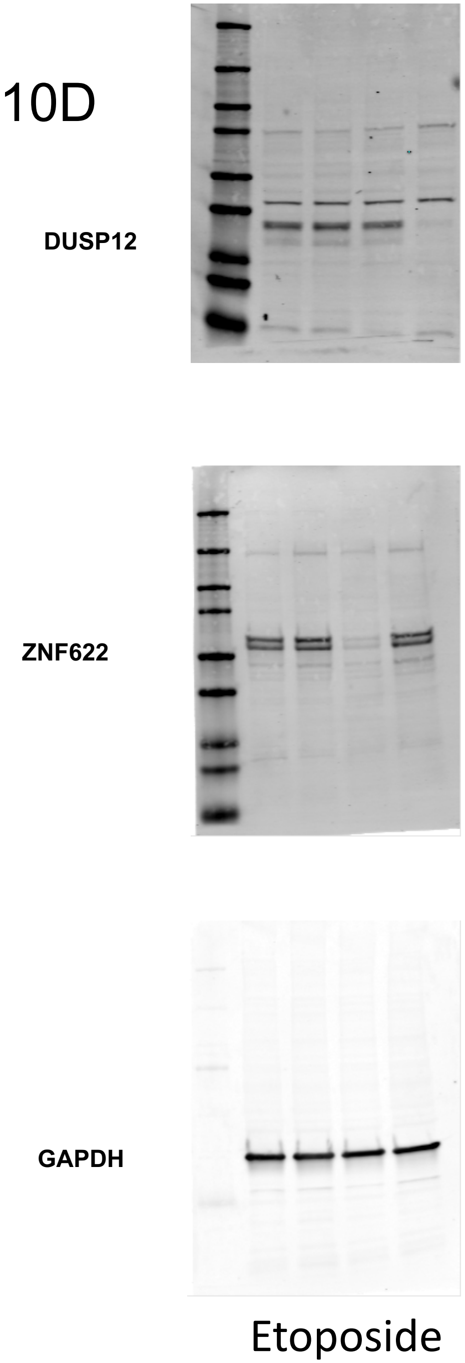

Fig. S10E

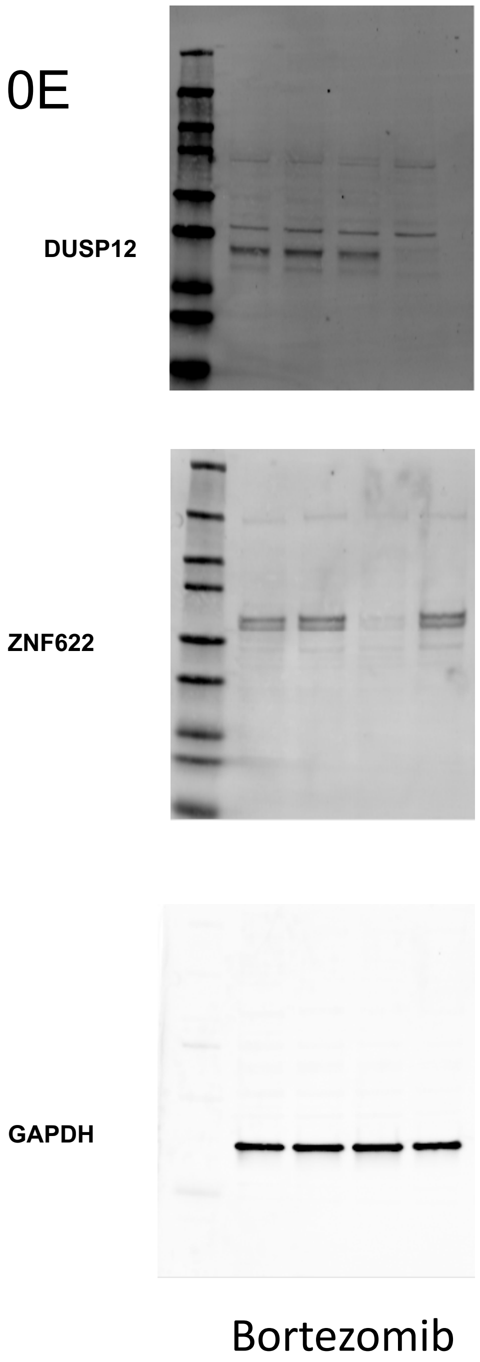

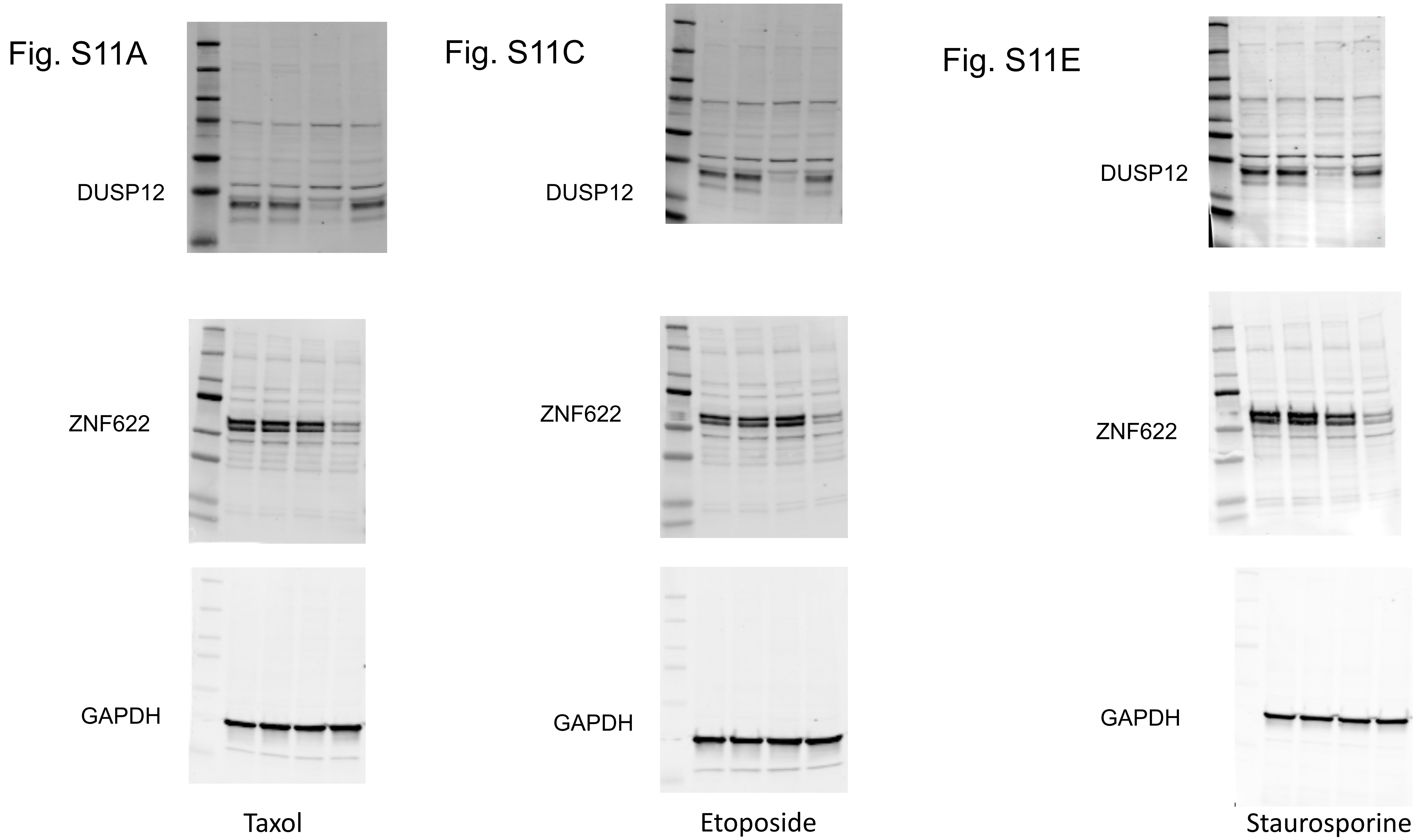
Fig. S11A
DUSP12
ZNF622
GAPDH
Taxol
Fig. S11C
DUSP12
ZNF622
GAPDH
Etoposide
Fig. S11E
DUSP12
ZNF622
GAPDH
Staurosporine

Fig. 13A

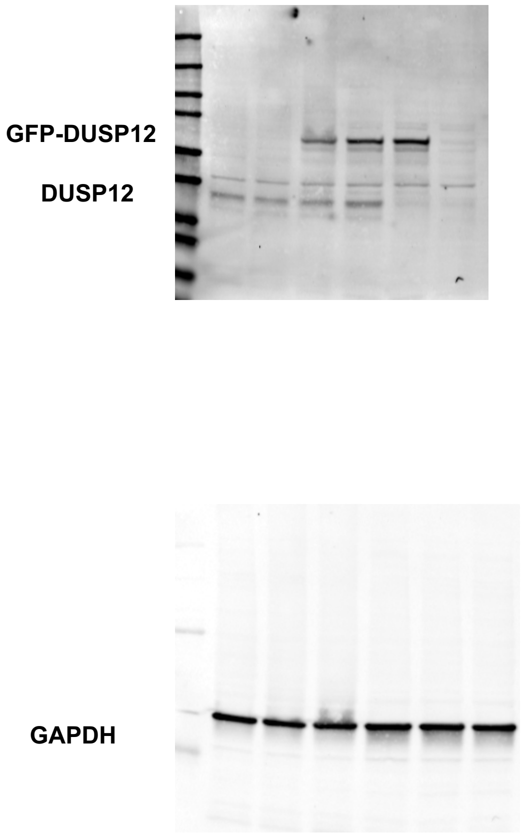

Fig. 13C

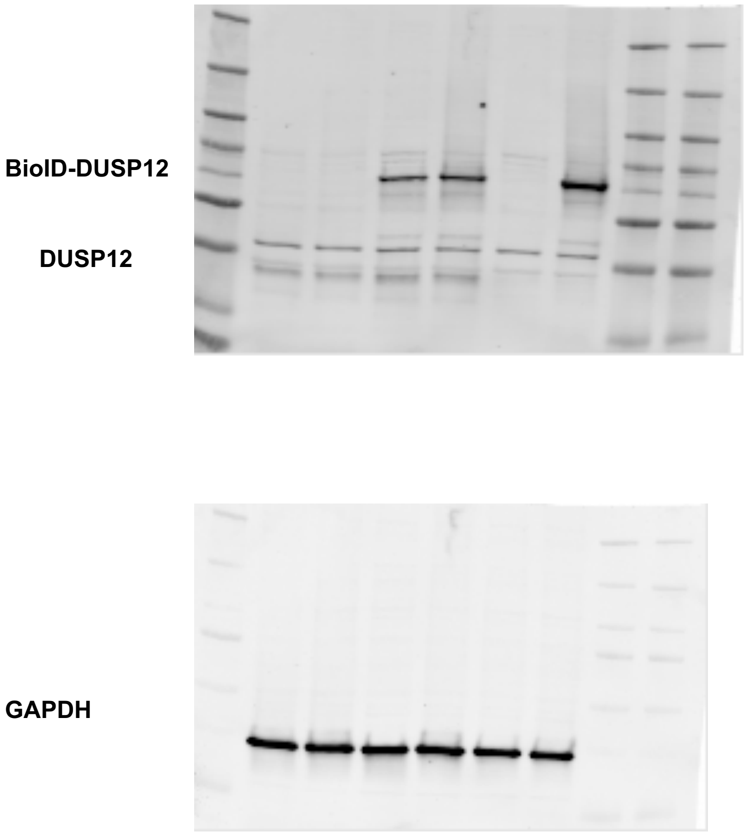

Fig. S14A

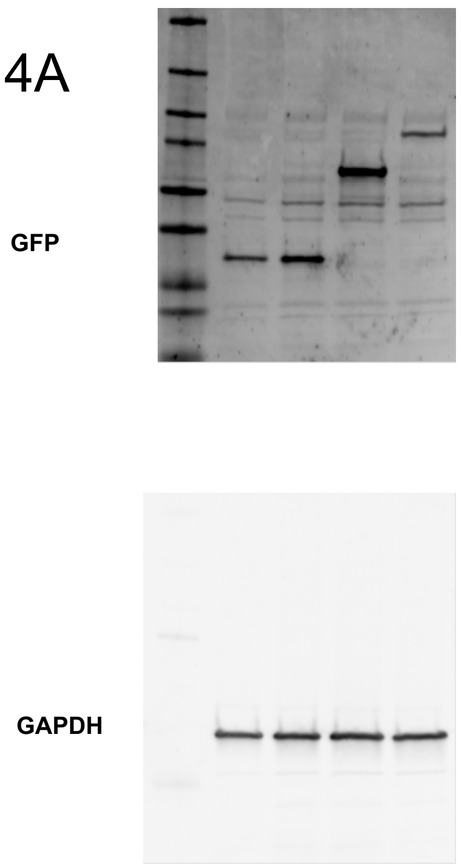

Taxol

Fig. S14B

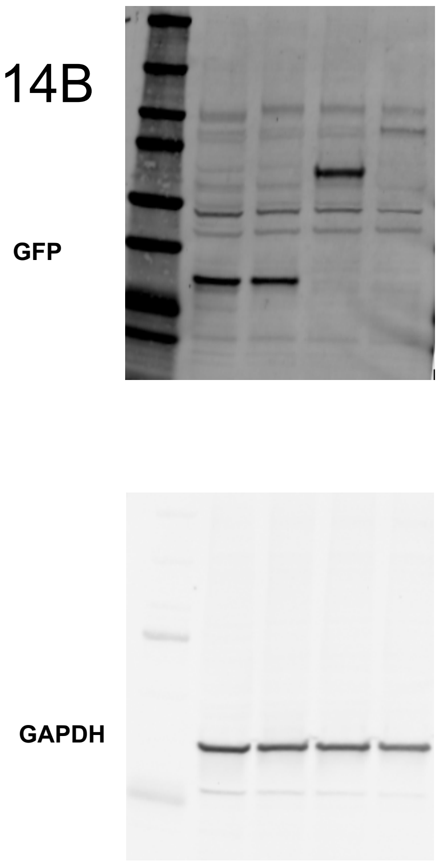

Staurosporine

Fig. S14C

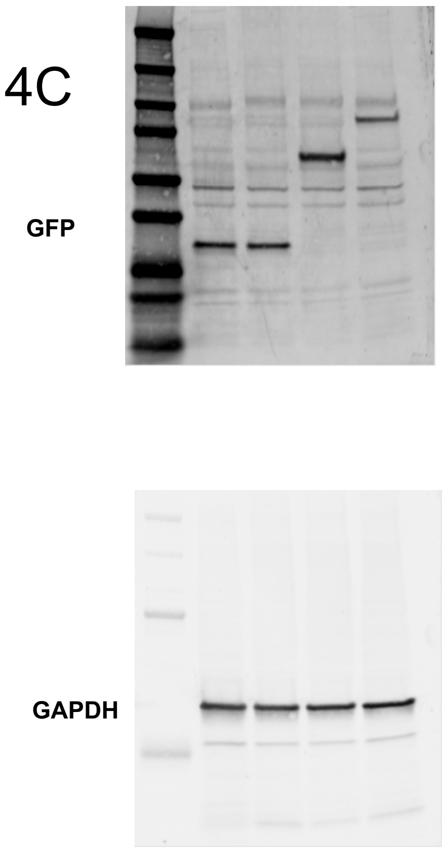

Colchicine

Fig. S14D

GFP

GAPDH

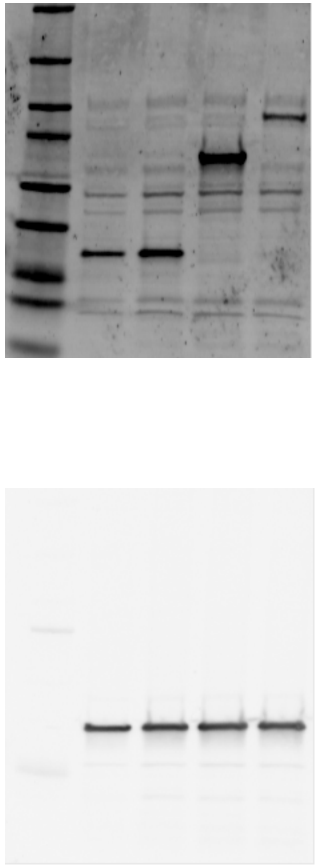

Etoposide

Fig. S14E

GFP

GAPDH

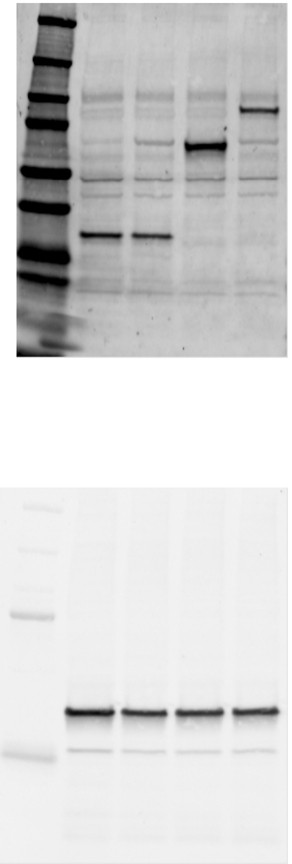

Bortezomib
