# Supplementary material for: DUSP12 promotes cell cycle progression and protects cells from ZNF622 mediated apoptosis
Source: Cell Death Dis. 2026 Mar 18;17(1):315. doi: 10.1038/s41419-026-08618-z (PMC13039736; doi:10.1038/s41419-026-08618-z)
Supplement: Supplementary file 4 — Table S2 [file 41419_2026_8618_MOESM4_ESM.pdf]

Table S2. List of key reagents and resources used in this study.

REAGENT or RESOURCE	SOURCE	IDENTIFIER
Antibodies		
Rabbit polyclonal anti-DUSP12	Proteintech	Cat# 15667-1-AP; RRID: AB_3085468
Mouse monoclonal anti-ZNF622	Santa Cruz Biotechnology	Cat# sc-100980; RRID: AB_2219571
Rabbit polyclonal anti-ZPR9 (ZNF622) Antibody	Bethyl Laboratories	Cat# A304-076A; RRID: AB_2621325
Rabbit polyclonal anti-HA	Proteintech	Cat# 51064-2-AP; RRID: AB_11042321
Mouse monoclonal anti-FLAG Dylight 800 Conjugated (clone 29E4.G7)	Rockland	Cat# 200-345-383; RRID: AB_10702994
Human polyclonal anti-Centromere Protein/CREST	Antibodies Incorporated	Cat# 15-234-0001; RRID: AB_2687472
Rat monoclonal anti-α-Tubulin (clone YOL1/34)	Bio-Rad	Cat# MCA78G; RRID: AB_325005
Rabbit polyclonal anti-phospho-Histone H3 (Ser10)	Millipore	Cat# 06-570; RRID: AB_310177
Goat polyclonal anti-Mad2 (C-19)	Santa Cruz Biotechnology	Cat# sc-6329; RRID: AB_648599
Mouse monoclonal anti-GAPDH (clone 1E6D9)	Proteintech	Cat# 60004-1-Ig; RRID: AB_2107436
Chicken polyclonal anti-GFP	Abcam	Cat# ab13970; RRID: AB_300798
Donkey polyclonal anti-Rat IgG (H+L), Cy3 AffiniPure	Jackson ImmunoResearch	Cat# 712-165-153; RRID: AB_2340667
Donkey polyclonal anti-Mouse IgG (H+L), Cy3 AffiniPure	Jackson ImmunoResearch	Cat# 715-165-151; RRID: AB_2315777
Donkey polyclonal anti-Human IgG (H+L), Cy5 AffiniPure	Jackson ImmunoResearch	Cat# 709-175-149; RRID: AB_2340539
Donkey polyclonal anti-Rat IgG (H+L), Cy5 AffiniPure	Jackson ImmunoResearch	Cat# 712-175-150; RRID: AB_2340671
Donkey polyclonal anti-Chicken IgY (IgG) (H+L), Fluorescein (FITC) AffiniPure	Jackson ImmunoResearch	Cat# 703-095-155; RRID: AB_2340356
Donkey polyclonal anti-Rabbit IgG (H+L), Fluorescein (FITC) AffiniPure	Jackson ImmunoResearch	Cat# 711-095-152; RRID: AB_2315776
Donkey polyclonal anti-Goat IgG (H+L), IRDye 680RD	LI-COR Biosciences	Cat# 926-68074; RRID: AB_10956736
Donkey polyclonal anti-Mouse IgG (H+L), IRDye 680RD	LI-COR Biosciences	Cat# 926-68072; RRID: AB_10953628
Donkey polyclonal anti-Mouse IgG (H+L), IRDye 800CW	LI-COR Biosciences	Cat# 926-32212; RRID: AB_621847
Donkey polyclonal anti-Chicken IgG (H+L), IRDye 800CW	LI-COR Biosciences	Cat# 926-32218; RRID: AB_1850023

Donkey polyclonal anti-Rabbit IgG (H+L), IRDye 680RD	LI-COR Biosciences	Cat# 926-68073; RRID: AB_10954442
Donkey polyclonal anti-Rabbit IgG (H+L), IRDye 800CW	LI-COR Biosciences	Cat# 926-32213; RRID: AB_621848
Chemicals, Peptides, and Recombinant Proteins		
Paclitaxel	Sigma-Aldrich	Cat# T7191; CAS: 33069-62-4
Staurosporine	Selleck Chemicals	Cat# S1421; CAS: 62996-74-1
Etoposide	Selleck Chemicals	Cat# S1225; CAS: 33419-42-0
Bortezomib	Selleck Chemicals	Cat# S1013; CAS: 179324-69-7
Colchicine	Selleck Chemicals	Cat# S2284; CAS: 64-86-8
Halt Protease Inhibitor Cocktail	Thermo Fisher Scientific	Cat# 87786
Halt Phosphatase Inhibitor	Thermo Fisher Scientific	Cat# 78429
Thymidine	Sigma-Aldrich	Cat# T1895; CAS: 50-89-5
Doxycycline	Sigma-Aldrich	Cat# D9891; CAS: 24390-14-5
MG132	Millipore Sigma	Cat# 474790; CAS: 133407-82-6
Hoechst 33342	Thermo Fisher Scientific	Cat# H1399; CAS: 23491-52-3
ProLong Gold Antifade Mountant	Thermo Fisher Scientific	Cat# P36934
Lipofectamine RNAiMAX	Thermo Fisher Scientific	Cat# 13778150
FuGENE HD Transfection Reagent	Promega	Cat# E2311
FuGENE 6 Transfection Reagent	Promega	Cat# E2691
S-protein Agarose	Millipore Sigma	Cat# 69704-4
Anti-FLAG M2 magnetic beads	Sigma-Aldrich	Cat# M8823
Biotin	Sigma-Aldrich	Cat# B4501-1G
Dynabeads MyOne Streptavidin C1	Thermo Fisher Scientific	Cat# 65002
Affi-Prep Protein A Resin	Bio-Rad	Cat# 156-0005
Critical Commercial Assays		
Gateway LR Clonase II Enzyme mix	Thermo Fisher Scientific	Cat# 11791020
Gateway BP Clonase II Enzyme mix	Thermo Fisher Scientific	Cat# 11789020
QuikChange Lightning Site-Directed Mutagenesis Kit	Agilent	Cat# 210518
QIAprep Spin Miniprep Kit	QIAGEN	Cat# 27106

PureYield Plasmid Midiprep System	Promega	Cat# A2495
SP6 TnT Quick Coupled Transcription/Translation System	Promega	Cat# L2080
Caspase-Glo 3/7 Assay System	Promega	Cat# G8091
MycoStrip Mycoplasma Detection Kit	InvivoGen	Cat# REP-MYS-10
eBioscience Annexin V Apoptosis Detection Kit	Thermo Fisher Scientific	Cat# 88-8006-74
Deposited Data		
Affinity-based mass spectrometry performed with LAP-DUSP12	This paper	
Proximity-based mass spectrometry performed with BioID2-DUSP12	This paper	
Experimental Models: Cell Lines		
HEPG2 cell line	ATCC	Cat# HB-8065; RRID: CVCL_0027
HeLa cell line	ATCC	Cat# CCL-2; RRID: CVCL_0030
HeLa FUCCI cell line	RIKEN BRC	Cat#; RRID: CVCL_VM98
HeLa-3XNLS-EBFP2	This paper	N/A
HeLa Flp-In T-Rex cell lines	Stephen Taylor Lab	(Tighe et al., 2004)
Inducible HeLa LAP-DUSP12 stable cell line	This paper	N/A
Inducible HeLa BioID2-DUSP12 stable cell line	This paper	N/A
Inducible HeLa LAP-DUSP12-C115A stable cell line	This paper	N/A
Inducible HeLa LAP-DUSP12-C115A stable cell line	This paper	N/A
Inducible HeLa LAP-DUSP12-R121A stable cell line	This paper	N/A
Inducible HeLa LAP-DUSP12-1-33aa stable cell line	This paper	N/A
Inducible HeLa LAP-DUSP12-1-168 stable cell line	This paper	N/A
Inducible HeLa LAP-DUSP12-34-168 stable cell line	This paper	N/A
Inducible HeLa LAP-DUSP12-34-340 stable cell line	This paper	N/A
Inducible HeLa LAP-DUSP12-169-340 stable cell line	This paper	N/A
Inducible HeLa LAP- ZNF622 stable cell line	This paper	N/A
Inducible HeLa LAP- ZNF622-rescue stable cell line	This paper	N/A
Inducible HeLa LAP- ZNF622-rescue-S143A stable cell line	This paper	N/A
Inducible HeLa LAP- ZNF622-rescue-S143D stable cell line	This paper	N/A

Inducible HeLa LAP-DUSP12-rescue stable cell line	This paper	N/A
Oligonucleotides		
siRNA Non-Targeting Control	Thermo Fisher Scientific	Cat# 4390844
siRNA against DUSP12	Thermo Fisher Scientific	Cat# 4390826; siRNA ID: s22244
siRNA against ZNF622 (ZPR9)	Thermo Fisher Scientific	Cat# 4392422; siRNA ID: s40388
siRNA against DUSP12	Thermo Fisher Scientific	Cat# 4390826; siRNA ID: s22245
Primer for cloning DUSP12-C115A: Fwd 5'-TGACTCCTGCATGAGCGTGCACCAACACCG-3’	Eurofins Genomics	N/A
Primer for cloning DUSP12-C115A: Rev 5'-CGGTGTTGGTGCACGCTCATGCAGGAGTCA-3’	Eurofins Genomics	N/A
Primer for cloning DUSP12-R121A: Fwd 5'-TTATGGCCACACTTGCACTGACTCCTGCAT-3’	Eurofins Genomics	N/A
Primer for cloning DUSP12-R121A: Rev 5'-ATGCAGGAGTCAGTGCAAGTGTGGCCATAA-3’	Eurofins Genomics	N/A
Primer for cloning DUSP12-1-33aa: Fwd 5'-GGGGACAAGTTTGTACAAAAAAGCAGGCTTCATGGGGTTGGAGGCTCCGGGCCCG -3’	Eurofins Genomics	N/A
Primer for cloning DUSP12-1-33aa: Rev 5'-GGGGACCACTTTGTACAAGAAAGCTGGGTCTCATCCTGGCTGCACTTCCAGC-3’	Eurofins Genomics	N/A
Primer for cloning DUSP12-1-168aa: Fwd 5'-GGGGACAAGTTTGTACAAAAAAGCAGGCTTCATGGGGTTGGAGGCTCGGGCCCG -3’	Eurofins Genomics	N/A
Primer for cloning DUSP12-1-168aa: Rev 5'-GGGGACCACTTTGTACAAGAAAGCTGGGTCTCACATTGCCTGGTATAATTTCAGTTGC -3’	Eurofins Genomics	N/A
Primer for cloning DUSP12-34-168aa: Fwd 5'-GGGGACAAGTTTGTACAAAAAAGCAGGCTTCATGGGGTGTATTTCGGTGGGGCCGCG-3’	Eurofins Genomics	N/A
Primer for cloning DUSP12-1-34-168aa: Rev 5'-GGGGACCACTTTGTACAAGAAAGCTGGGTCTCACATTGCCTGGTATAATTTCAGTTGC -3’	Eurofins Genomics	N/A
Primer for cloning DUSP12-34-340aa: Fwd 5'-GGGGACAAGTTTGTACAAAAAAGCAGGCTTCATGGGGTGTATTTCGGTGGGGCCGCG-3’	Eurofins Genomics	N/A

Primer for cloning DUSP12-34-340aa: Rev 5'-GGGGACCACTTTGTACAAGAAAGCTGGGTCTCATATTTTTCCTGTTTGTGATCCCAAACAG-3’	Eurofins Genomics	N/A
Primer for cloning DUSP12-169-340aa: Fwd 5'-GGGGACAAGTTTGTACAAAAAAGCAGGCTTCATGGGGATACGAAGTGGATACCTCTAGTGCAA -3’	Eurofins Genomics	N/A
Primer for cloning DUSP12-169-340aa: Rev 5'-GGGGACCACTTTGTACAAGAAAGCTGGGTCTCATATTTTTCCTGTTTGTGATCCCAAACAG-3’	Eurofins Genomics	N/A
Primer for cloning ZNF622 S143A: Fwd 5'-CCAGCCGTCCATGGCTCCCAAGAAGGC-3’	Eurofins Genomics	N/A
Primer for cloning ZNF622 S143A: Rev 5'-GCCTTCTTGGGAGCCATGGACGGCTGG -3’	Eurofins Genomics	N/A
Primer for cloning ZNF622 S143D: Fwd 5'-CCCAGCCGTCCATGGATCCCAAGAAGGCGC -3’	Eurofins Genomics	N/A
Primer for cloning ZNF622 S143D: Rev 5'-GCGCCTTCTTGGGATCCATGGACGGCTGGG -3’	Eurofins Genomics	N/A
Primer for cloning ZNF622-Rescue: Fwd 5'-GACCTGGATGGCGACGATTGGGAGGACATAGATTCTGAT-3’	Eurofins Genomics	N/A
Primer for cloning ZNF622-Rescue: Rev 5'-ATCAGAATCTATGTCCTCCCAATCGTCGCCATCCAGGTC-3’	Eurofins Genomics	N/A
Primer for cloning DUSP12-Rescue: Fwd 5'-CGCATTCCAAATACATAAGAATAGAGTGGATGAAATG -3’	Eurofins Genomics	N/A
Primer for cloning DUSP12-Rescue: Rev 5'-GGTGTGATCCACCTACCACAAGAGCACTG -3’	Eurofins Genomics	N/A
Recombinant DNA		
pDONR221-DUSP12	DNASU Plasmid Repository	Clone ID: HsCD00041750
pLX304-ZNF622 (ZPR9)	DNASU Plasmid Repository	Clone ID: HsCD00441669
pGLAP1-DUSP12	This paper	N/A
pGLAP1-DUSP12-C115A	This paper	N/A
pGLAP1-DUSP12-R121A	This paper	N/A
pGLAP1-DUSP12-1-33aa	This paper	N/A
pGLAP1-DUSP12-1-168aa	This paper	N/A
pGLAP1-DUSP12-34-168aa	This paper	N/A
pGLAP1-DUSP12-34-340aa	This paper	N/A
pGLAP1-DUSP12-169-340aa	This paper	N/A
pGBioID2-DUSP12	This paper	N/A
pCS2-HA-DUSP12	This paper	N/A
pCS2-HA-DUSP12	This paper	N/A
pCS2-HA-DUSP12-C115A	This paper	N/A
pCS2-HA-DUSP12-R121A	This paper	N/A
pCS2-HA-DUSP12-1-33aa	This paper	N/A

pCS2-HA-DUSP12-1-168aa	This paper	N/A
pCS2-HA-DUSP12-34-168aa	This paper	N/A
pCS2-HA-DUSP12-34-340aa	This paper	N/A
pCS2-HA-DUSP12-169-340aa	This paper	N/A
pGLAP1-ZNF622	This paper	N/A
pGLAP1-ZNF622-S143A	This paper	N/A
pGLAP1-ZNF622-S143D	This paper	N/A
pGLAP1-ZNF622-rescue	This paper	N/A
pGLAP1-ZNF622-rescue-S143A	This paper	N/A
pGLAP1-ZNF622-rescue-S143D	This paper	N/A
pCS2-Flag-ZNF622	This paper	N/A
pGLAP1-GFP	This paper	N/A
pCS2-HA-GFP	This paper	N/A
pGLAP1-DUSP12-rescue	This paper	N/A
pGLAP1-DUSP12-rescue	This paper	N/A
BioID-DUSP12-rescue	This paper	N/A
Software and Algorithms		
GraphPad Prism 5	GraphPad	RRID: SCR_002798
BioRender	BioRender	RRID: SCR_018361
Modfit	Cytonome Verity	RRID: SCR_016106
FlowJo v11.0	FlowJo LLC	RRID: SCR_008520
